# SERS Facemask for Rapid and Portable Sensing Mycobacterium Tuberculosis Antigens for TB Screening

**DOI:** 10.1002/advs.202523921

**Published:** 2026-06-19

**Authors:** Lingzhi Chen, Jiaqi Yu, Jing Xu, Lingqi Chen, Ninghao Zhang, Wenrui Li, Runmin Zeng, Xiaomin Luo, Yiqun Chang, Jiayin Lai, Wenshi Huang, Xiaochen Liang, Ting Zhao, Shanze Chen, Huaihong Cai, Yanguang Cong, Pinghua Sun, Jiang Pi, Xueqin Huang, Haibo Zhou, Junxia Zheng

**Affiliations:** ^1^ State Key Laboratory of Bioactive Molecules and Druggability Assessment Guangdong Basic Research Center of Excellence for Natural Bioactive Molecules and Discovery of Innovative Drugs The Fifth Affiliated Hospital College of Pharmacy Jinan University Guangzhou China; ^2^ The First Dongguan Affiliated Hospital Guangdong Provincial Key Laboratory of Medical Immunology and Molecular Diagnostics School of Medical Technology Guangdong Medical University Dongguan China; ^3^ The Ninth People's Hospital of Dongguan Dongguan China; ^4^ College of Chemistry and Materials Science Jinan University Guangzhou China; ^5^ Institute for Safflower Industry Research Key Laboratory of Xinjiang Phytomedicine Resource and Utilization Ministry of Education School of Pharmacy Shihezi University Shihezi China; ^6^ School of Biomedical and Pharmaceutical Sciences Guangdong University of Technology Guangzhou China

**Keywords:** facemask, liquid biopsy, point‐of‐care testing, surface‐enhanced Raman scattering, tuberculosis

## Abstract

Rapid and sensitive diagnostic strategies are crucial for the prevention and control of tuberculosis (TB). Unlike the invasive TB diagnostic strategy in the clinic, here we introduce a convenient, portable, and non‐invasive system that is constructed by Ag@Au nanoflower (NF) array‐based sensing facemask and catalytic/plasmonic urchin‐shaped Au─Ag embedded covalent organic framework (U@COF) sensor. This system detects TB antigen ESAT‐6/CFP‐10 complex in droplet or sputum samples from a variety of clinical settings. Practical analysis of clinical samples demonstrates this assay is capable of classifying the negative (*N = 12*) and positive (*N = 17*) TB patients with satisfactory sensitivity (76.5%) and specificity (100%), among whom two TB‐infected patients (TB3 and TB5) missed by droplet analysis are successfully identified by sputum analysis. More importantly, two cases with abnormally elevated ESAT‐6/CFP‐10 levels are screened out from close contacts of TB patients (*N = 6*), which is highly suggestive of TB infection (Close contacts 3 and 4). This portable mask is suitable for rapid diagnosis of TB infection in patients with cough, particularly for screening of TB close contacts.

## Graphical abstract

Our study introduced an Au─Ag embedded covalent organic framework (U@COF) ‐mediated facemask for sensing TB antigen ESAT‐6/CFP‐10 complex in clinical droplet samples toward TB screening. Practical analysis of clinical samples demonstrated the availability of our facemask, which is capable of identifying the TB subjects (*N = 17*) from healthy candidates (*N = 12*), and screening TB close contacts (*N = 6*), with satisfactory sensitivity (76.5%) and specificity (100%). Unlike traditional TB diagnostic tests (smears, Xpert, TST), our proposed mask can quickly, simply, and non‐invasively detect and screen the early infection of TB with great patient compliance.

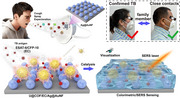

## 1 Introduction

Tuberculosis (TB) is a respiratory infectious disease induced by *Mycobacterium tuberculosis* (MTB) with significant morbidity and mortality [1]. According to the “Global Tuberculosis Report 2025” released by the World Health Organization (WHO), the global death toll from TB was 1.23 million (1.13–1.33 million) in 2024, with a mortality rate of 11.5% [2, 3]. The conventional culture test remains the gold standard for TB diagnosis but requires a turnaround time of 3–4 weeks, making it unsuitable for early clinical screening [4]. Current TB screening methods, including the tuberculin skin test (TST) and the interferon gamma release test (IGRAs), are invasive analytical approaches with poor patient compliance and low sensitivity/specificity [5, 6, 7]. Polymerase Chain Reaction (PCR)‐based GeneXpert offers relatively higher sensitivity and specificity for TB, while suffering from high cost, tedious sample preparation, and the need for expensive equipment [8]. Especially, TB is mainly transmitted through the respiratory tract, exhaled samples (e.g., droplets and expectoration) contain plenty of TB target biomarkers, especially in the early stage of infection [9, 10]. Therefore, developing a simple, portable, non‐invasive, and rapid exhaled substrate‐based detection technology can efficiently reveal the infection status of TB, enabling early screening and more effective control of TB.

The facemask device, as a wearable, non‐intrusive, and onsite sampler, opens up a novel avenue for the direct detection of various exhaled substances [11, 12]. By embedding the sensing substrate into the breathing area, the mask can continuously capture and detect aerosols, volatile organic compounds (VOCs), and droplets released by the wearer's exhalation, sneezing, coughing, and other behaviors. So far, the application of facemasks in monitoring a variety of diseases, including viruses, asthma, lung cancer, and chronic obstructive pulmonary disease, has also been extensively reported [13, 14, 15, 16]. Despite this promise, however, fewer attempt made to develop a facemask sensor for the diagnosis and monitoring of TB. One of the reasons is mainly due to the fast emission velocity of droplets or expectoration from the patients (2–10 m/s) [13, 17], making it difficult to effectively capture and absorb the target biomarkers. Although studies have revealed that modulating the surface area and surface energy of sensing materials can increase the retention rate of molecules [18], the mass transfer behavior of gas‐liquid molecules remains elusive in most cases. Another reason is the relatively ultralow amounts of MTB biomarkers in droplets or expectoration compared to other liquid biopsies, along with interindividual variability, severely weakening the sensitivity and accuracy of testing. Furthermore, the binding affinity between analytes and biometric elements is adversely affected by the high abundance of interfering molecules.

To realize sensitive and reliable detection of low‐abundance biomarkers in exhaled samples, Surface‐enhanced Raman spectroscopy (SERS) has emerged as a technique offering high sensitivity, rapid response, and abundant “fingerprint” information on analytes [19, 20, 21, 22]. The construction of high‐intensity local electromagnetic fields through well‐designed plasmonic nanostructures (e.g., Au, Ag nanoparticles (NPs)) significantly enhances the Raman scattering of analytes (10^8^–10^10^) [23]. Besides, some SERS active substrates, such as Cu_2_O, WO_3_, MoS_2_, and VO_2_, can also amplify the SERS signal through the strong charge transfer at the substrate‐analyte contact area by chemical mechanism (CM) enhancement [24, 25, 26]. Covalent organic framework (COF) is a new type of SERS sensor, which is a porous nanocomposite composed of organic components [27, 28, 29]. The large surface area and rich conjugated system provide abundant binding sites for target molecules. By adjusting the monomer composition and polymerization mode, COF exhibits terrific SERS enhancement capability with the intrinsic Raman peak [30, 31, 32]. This “label‐free” strategy not only omits the tedious functionalization procedures of the Raman reporter, but also eliminates the issue of nonuniform distribution of reporting molecules on the substrate. Except for SERS properties, COF with enzyme‐mimicking function can be expanded to a catalytic colorimetric assay [33, 34, 35]. Therefore, the multifunctional COF with the inherent characteristics of catalytic colorimetry and SERS can perform multimode sensing.

**SCHEME 1:**
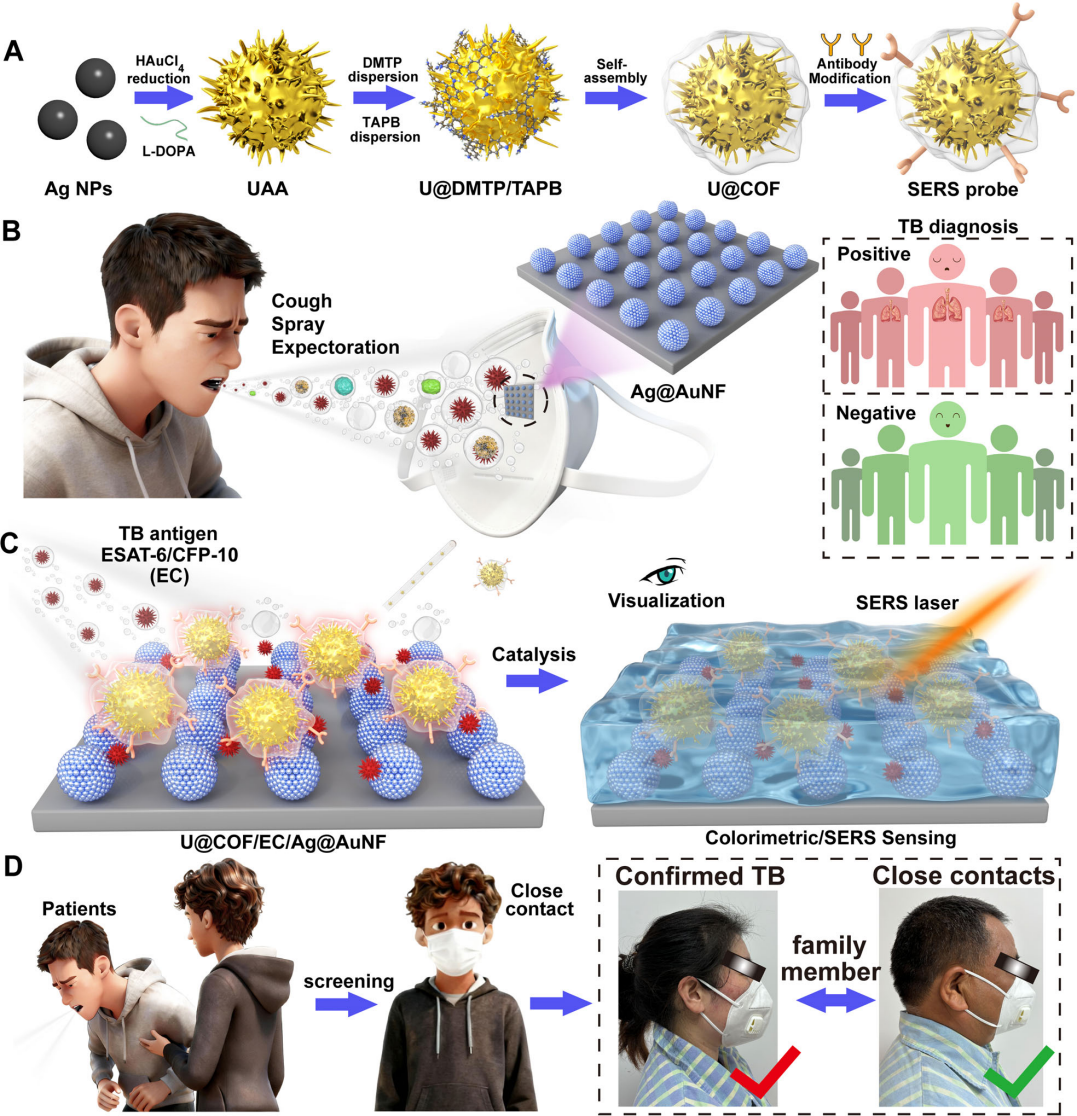
(A) Schematic illustration of the fabricated process for U@COF (SERS probe). (B) Principle of facemask sensor for detection of TB antigen ESAT‐6/CFP‐10 complex toward TB diagnosis. (C) Principle of colorimetric/SERS dual‐sensing ESAT‐6/CFP‐10 complex. (D) Facemask sensor for screening TB close contact.

In this work, we report a portable facemask that is equipped with a highly adsorptive Ag@Au nanoflower (NF) array composite SERS chip on the inner fabric layer covering the filtering holes, enabling for continuous collection of the respiratory secretions released by the patient during coughing or expectoration without affecting breathing. By wearing the facemask, the virulence factor *MTB*‐derived CFP‐10 and ESAT‐6 antigen complex (ESAT‐6/CFP‐10) that was secreted during the early stage of TB infection can be directly detected from droplets or expectoration (Scheme 1B) [36, 37]. Additionally, a urchin‐shaped Au─Ag embedded COF (U@COF) sensor was designed to realize colorimetric/SERS dual‐mode sensing of ESAT‐6/CFP‐10 in complex clinical samples (Scheme 1A). In the presence of the target ESAT‐6/CFP‐10 (EC), an Ag@AuNF array with high surface energy allowed the efficient adsorption and deposition of the analytes, followed by introducing a U@COF sensor to form U@COF/EC/Ag@AuNF satellite nanoassemblies (Scheme 1C), where U@COF with strong SERS activity further amplified the SERS effect of the Ag@AuNF array for ultra‐sensitive detection of ESAT‐6/CFP‐10. After the catalytic reaction, the colorimetric mode is easy to operate and convenient for observation, making it suitable for on‐site Proof of Concept (POC) testing. Diagnostic performance was further assessed in clinical samples collected from infected or uninfected TB patients, and the results were compared to clinical information. Unlike the traditional TB diagnosis tests (smears, Xpert, TST), our facemask can rapidly, facilely, and non‐invasively detect the occurrence and development of TB infection with great patient compliance. Importantly, based on their portable and wearable performance, this facemask sensor held great potential to screen close contacts of TB (Scheme 1D), allowing for the early warning and diagnosis of TB in advance.

## 2 Materials and Methods

### 2.1 Materials and Reagents

Hydrogen tetrachloroaurate (HAuCl_4_·3H_2_O), 2,5‐dimethoxyp‐benzaldehyde (DMTP), 1,3,5‐tris (4‐aminophenyl) benzene (TAPB), ascorbic acid (AA), trisodium citrate, silver nitrate (AgNO_3_), acetonitrile (ACN), Tetramethylbenzidine (TMB), 4‐mercaptophenylboronic acid (4‐MPBA), L‐dopamine (L‐DOPA), and phosphate‐buffered saline (PBS) were supplied from Macklin (Shanghai, China). Butanol, acetone, acetic acid (HAc), polyvinylpyrrolidone powders (PVP), sodium phosphate dibasic (Na_2_HPO_4_), and sodium dihydrogen phosphate anhydrous (NaH_2_PO_4_) were purchased from Aladdin Biochem Technology Co., Ltd. (Shanghai, China). All chemicals were analytical grade and used without further purification. ESAT‐6/CFP‐10 antigen complex was obtained from GeneOptimal Co., Ltd. (Shanghai, China), and anti‐CFP‐10 rabbit antibody (Ab45073) was purchased from Abcam (Cambridge, UK). ESAT‐6/CFP‐10 aptamer (5’‐SH‐GCCTGTTGTGAGCCTCCTAACCCCATCTTATACGT ATATGGACTCATCTCGACCCCCGATAGGCTTGGTACATGCTTATTCTTGTCTCCC‐3’) was synthesized by Sangon Biotechnology Co. Ltd (Shanghai, China). The samples were recruited and taken by the Dongguan Ninth People's Hospital (Dongguan, China).

### 2.2 Synthesis of U@COF

U@COF was prepared in two‐step processes as depicted in Scheme 1A, including the formation of urchin‐shaped Au─Ag alloy (UAA) NPs and COF‐coated UAA (U@COF). First, UAA NPs were synthesized based on a seed growth approach with some modifications [38]. Briefly, 9 mL of Ag NO_3_ solution was preheated to boil with stirring. During the procedure of heating, 1% trisodium citrate was added to the system under stirring, and the color of the solution turned transparent to yellowish‐green, indicating the formation of Ag NPs. Then, the resultant Ag NPs were mixed with an aqueous solution containing 9.6 mL HAuCl_4_ and 9.6 mL L‐Dopa (10 mm) with stirring at 15°C. After sonication for 30 min, the obtained UAA NPs were collected by centrifugation, washed three times with deionized water, and redispersed in ethanol until future use.

For the synthesis of U@COF, 25 mm of DMTP and 30 mm TAPB were dissolved in an acetone solution to prepare precursor solutions, respectively [39]. According to the experimental requirements, acetone can be replaced with butanol or ACN for dispersion. Then, 1 mL of freshly prepared UAA NPs was mixed with 200 µL of acetic acid and thoroughly oscillated at room temperature for 24 h. The dark‐green product formed at the end of the reaction indicated the successful generation of U@COF. Finally, the supernatant was removed, and the deposit was collected by centrifugation, washed with deionized water several times, and stored at 4°C for future use. The U@COF with different thicknesses of COF shell was prepared by controlling the amount of DMTP and TAPB at different volumes (0.1, 0.2, 0.3, 0.5, 0.7, and 1 mL). COF was self‐assembling under similar reaction conditions, except for the absence of UAA. To prepare the antibody‐modified U@COF, CFP‐10 antibody (5 µL, 1 mg/mL) was mixed with 10 µL of 0.1 wt.% EDC/NHS for 1 h to activate carboxyl groups. The mixture was then incubated with 1 mL of U@COF with gentle shaking overnight. The activated carboxyl group on the antibody further reacted with the amino group on the COF by the amide bond, thereby forming antibody‐modified U@COF.

### 2.3 Self‐Assembly of Ag@AuNF for Preparing an Array

Ag@AuNF array was prepared in three consecutive stages (Scheme 1B), including the formation of Au nanocages (NCs) cores, the reduction of Ag@AuNF, and the arrangement of the array. For the synthesis of Au NCs, the solution of glycol (6 mL) was heated to 150°C under the oil bath for 1 h under gentle stirring. Subsequently, 120 µL of Na_2_S (3 mm) was added to the above solution and reacted for 9 min. Thereafter, 1.5 mL of PVP and 0.5 mL of AgNO_3_ (282 mm) were rapidly injected into the reaction solution, respectively, and stirred for 18 min. Then, the mixture was placed in an ice water bath to cool down, during which Ag grew to form the Au NCs. The resulting samples were collected via centrifugation, washed once with acetone, twice with deionized water, and stored at 4°C for future use.

For the synthesis of Ag@AuNF, 100 mg of AA and 66.6 mg of PVP were dispersed in 13.5 mL of ultrapure water with stirring. 0.5 mL of as‐prepared Ag NCs solution and 0.6 mL of NaOH solution (0.2 m) were added into the mixed solution, followed by the dropwise addition of aqueous HAuCl_4_ solution (5 mm, 2 mL) using a syringe pump at an injection rate of one drop every 30 s. The reaction was maintained for 30 min after injection. The resultant Ag@AuNF was collected by centrifugation, purified by ethanol, followed by water, and stored at 4°C for future use.

Ag@AuNF was self‐assembled at the water‐hexane interfacial system to form a close‐packed array [40]. First, 6 mL of Ag@AuNF was added to the clean glass beaker. Then, hexane (2 mL) was added to the mixture successively, followed by standing for 10 min to form an immiscible liquid system. Next, ethanol (1.5 mL) as an inducer was added to the mixed solution via a syringe, which made Ag@AuNF to form a monolayer at the water‐hexane interface by lowering the interfacial interface. Then, a clean silicon wafer was immersed in the solution at a small angle and then pulled it out slowly along the liquid surface. Finally, the closely arranged Ag@AuNF was transferred onto silicon wafers air dried at room temperature. To selectively capture ESAT‐6/CFP‐10, SH‐Apt was denatured and annealed (95°C, 10 min) before use. Then, 50 µL SH‐Apt (10 µm) was added to the Ag@AuNF array and incubated for 4 h. SH‐Apt was modified to the surface of the Ag@AuNF array by Au─S bond.

### 2.4 Characterization and Measurement

The morphology and element composition of U@COF and Ag@AuNF were characterized by field‐emission transmission electron microscopy (TEM) (JEM‐2100F, Japan) equipped with EDX. The surface properties of the U@COF were further measured by x‐ray photoelectron spectroscopy (XPS) (PHI 5000 Versa Probe, USA). The morphologies of the Ag@AuNF array were characterized via scanning electron microscopy (SEM) using an S‐4800 instrument (JSM‐7500F, Japan). The absorption spectra were assessed by using Ultraviolet‐Visible (UV–vis) spectrophotometer (Shimadzu UV‐3600, Japan). The contact angles (CAs) of water were calculated on the DSA10 MK2 Drop Shape Analyzer (Krüss, Germany) at room temperature. All the SERS spectra were conducted by a Raman microscope (LabRAM HR, Japan) under the monochromatic excitation laser at 633 nm laser wavelength, with a 50 × telephoto objective at a power density of 16.0 mW/µm^2^, and 2s of acquisition time. The mimetic peroxidase activity of U@COF was determined using the TMB colorimetric system. After U@COF attaching to the array, the PBS buffer (pH = 4) containing 20 µL of H_2_O_2_ (1 m) and 200 µL of TMB was added into the array and incubated for 15 min. The absorbance of oxTMB at 652 nm was recorded. To effectively capture exhaled substances, the Ag@AuNF array was cut into small pieces and attached to the breathing filter of a commercial mask with medical tape.

### 2.5 Detection of ESAT‐6/CFP‐10 in Ex Vivo

Typically, ESAT‐6/CFP‐10 solution (10 µL) with different concentrations from 2 to 200 ng/mL was dropped and fully dried on the Apt‐modified Ag@AuNF‐embedded facemask. Then, the antibody‐decorated U@COF substrate was added into the chip, so that U@COF can capture the ESAT‐6/CFP‐10 antigen complexes and attach onto the surface of the chip. After that, the chip was washed with PBS, and the SERS signal of COF was obtained. Furthermore, we used commercial sprayers to simulate the emission of droplets during human coughing.

Different carrier gas flow rates (50–200 sccm) were controlled by flow controllers, while the distance between the SERS substrate and the nozzle was maintained within 25 cm. The nebulizing time was 3 s each time. ESAT‐6/CFP‐10 with respective concentrations was spiked to human saliva or sputum prior to nebulizing to emulate the detection of respiratory excretion solution. The control sample refers to the respiratory excretion solution mixed with the PBS buffer without any ESAT‐6/CFP‐10 target.

Diverse biologically relevant interferents, such as NaCl, Na_2_CO_3_, NaH_2_PCO_4_, K_2_HPCO_4_, and NaHSO_3_ were added into the solution to evaluate the anti‐interference of the assay. Furthermore, a series of proteins, such as MTB antigen Ag85B, alpha fetoprotein (AFP), lipoarabinomannan (LAM), prostate specific antigen (PSA), and Lipopolysaccharide (LPS) were added into the solution to estimate the specificity of the assay. These samples were measured by the SERS assay and compared to a noninterfering sample to reveal their performance. Uniformity was estimated by monitoring 30 random regions of the facemask chip. The above chip was stored in the dark at 4°C and room temperature for different days, and then conducted to ESAT‐6/CFP‐10 assay to investigate the stability. Each SERS or absorption spectrum represented the average value of three data. The limit of detection (LOD) was defined as the ratio of the standard deviation of the blank to the slope of the linear equation of the calibration plot (LOD = 3 SD/slope, *n* = 3).

### 2.6 In Vivo Assay of SERS Facemask in Real Samples

To evaluate the clinical applicability of the facemask sensor for TB diagnosis, the confirmed patients (*n* = 17), non‐TB infected patients (*n* = 12), and close contacts (*n* = 6) were recruited by Dongguan Ninth People's Hospital (Dongguan Infectious Disease Hospital). Respiratory specimens were analyzed using concentrated smear microscopic examination of acid‐resistant bacilli, PCR‐based Xpert assay, and TST reaction. The participants were classified as “confirmed TB” and “non‐TB” groups. Close contacts, regardless of whether they showed symptoms of TB or not, all met the recruiting criteria for the “Close contacts” group. In general, if the patient presented the following two criteria: TB‐related symptoms/signs (Coughing for more than 2 weeks, hemoptysis, fever, night sweats, weight loss, etc.), abnormal TB chest x‐rays, and positive immunologic evidence (i.e., positive TST), it can be determined as positive. All procedures involving human samples were conducted in a biosafety cabinet located in a Biosafety Level 2 (BSL‐2) laboratory.

Based on the clinical diagnostic data, SERS or colorimetric signals were obtained from the facemask, and the concentrations of ESAT‐6/CFP‐10 in samples were quantified using the established standard curve. Given that the significant influence of viscosity on the testing, the viscous sputum samples were diluted and lysed by digestant‐decontaminant solution (containing 25 mL of 4% NaOH, 25 mL of 2.9% sodium citrate, and 0.25 g of N‐acetyl‐L‐cysteine (NALC)) at room temperature for 15 min with gentle shaking before measurement. Air droplets were tested directly after air‐drying.

For the investigation of wearing time, a TB patient with coughing wore a mask that contained the SERS chip. The chip was removed from the mask, and the SERS signal was measured every 5 min. The ESAT‐6/CFP‐10 concentration at different sampling time points was analyzed by the SERS signal. Considering coughing is an involuntary reflex caused by irritation of the respiratory mucosa, the different cough times were collected from TB patients. When the number of coughs fell within intervals, the chip was removed from the mask, and SERS measurement was performed. For the investigation of patient compliance, we mainly inquiry patients about their comfortability and acceptability (sensitivity, specificity, and pain tolerance) among the current mainstream Xpert, smear, TST test, and the SERS mask.

### 2.7 Statistical Analysis

All experiments were carried out at least in triplicate (n≧3) and all data presented as mean ± S.D. GraphPad Prism 10.2.2 software was used for graphical representation and statistical analysis. Statistical significance (*p*‐value) was assessed using ANOVA analysis and by independent *t*‐tests for pairwise comparisons, ^*^ *p* < 0.05;^**^ *p* < 0.01; ^***^ *p* < 0.001. Each image involved in visual inspection in the figure is a representative of at least three independent experiments. The study was conducted in accordance with clinical guidelines and approved by the local Bio‐Ethics Committee (YJYS202407001). All patients were informed and consented to provide experimental samples. Based on the clinical dataset, receiver operating characteristic (ROC) curve analysis was conducted to assess the diagnostic threshold. The optimal cutoff value was determined by the Youden index (Youden index = Sensitivity + Specificity‐1). The samples exhibited signal intensities above this threshold, classified as positive, and those below it as negative. *pROC* was calculated by the SDSS software and typically used to verify whether the AUC value is significantly higher than 0.05. Typically, *pROC* < 0.05 usually indicates that the discriminative ability (diagnostic efficacy) of the current model is statistically significant. In a word, the ROC curve is used to evaluate the performance of model classification, while the *pROC* determines whether the model is reliable.

## 3 Results and Discussion

### 3.1 Synthesis and Characterization of U@COF

Herein, we developed a novel COF‐based SERS substrates (U@COF) that was made in an urchin‐shaped Au/Ag (UAA) plasmonic core and porous COF shell with high dispersibility and colloidal stability. The stepwise synthesis of the U@COF structure was depicted in Scheme 1A. Initially, UAA was synthesized through the seed‐mediated growth and in situ reduction reactions, where the typical sharp tentacles and huge surface areas allowed the formation of a strong and highly dense “hot spot” area. And subsequently, TAPB and DMTP monomers self‐assembled on the surface of UAA through Schiff base reaction to form a COF layer, the rich active sites and positive charge of which were conducive to the highly efficient conjugation of metal NPs. The COF layer introduced in this study not only provided a protective layer for maintaining the stability and monodispersity of UAA but also endowed the probe with strong plasma coupling and nanoenzyme catalytic activity.

**FIGURE 1:**
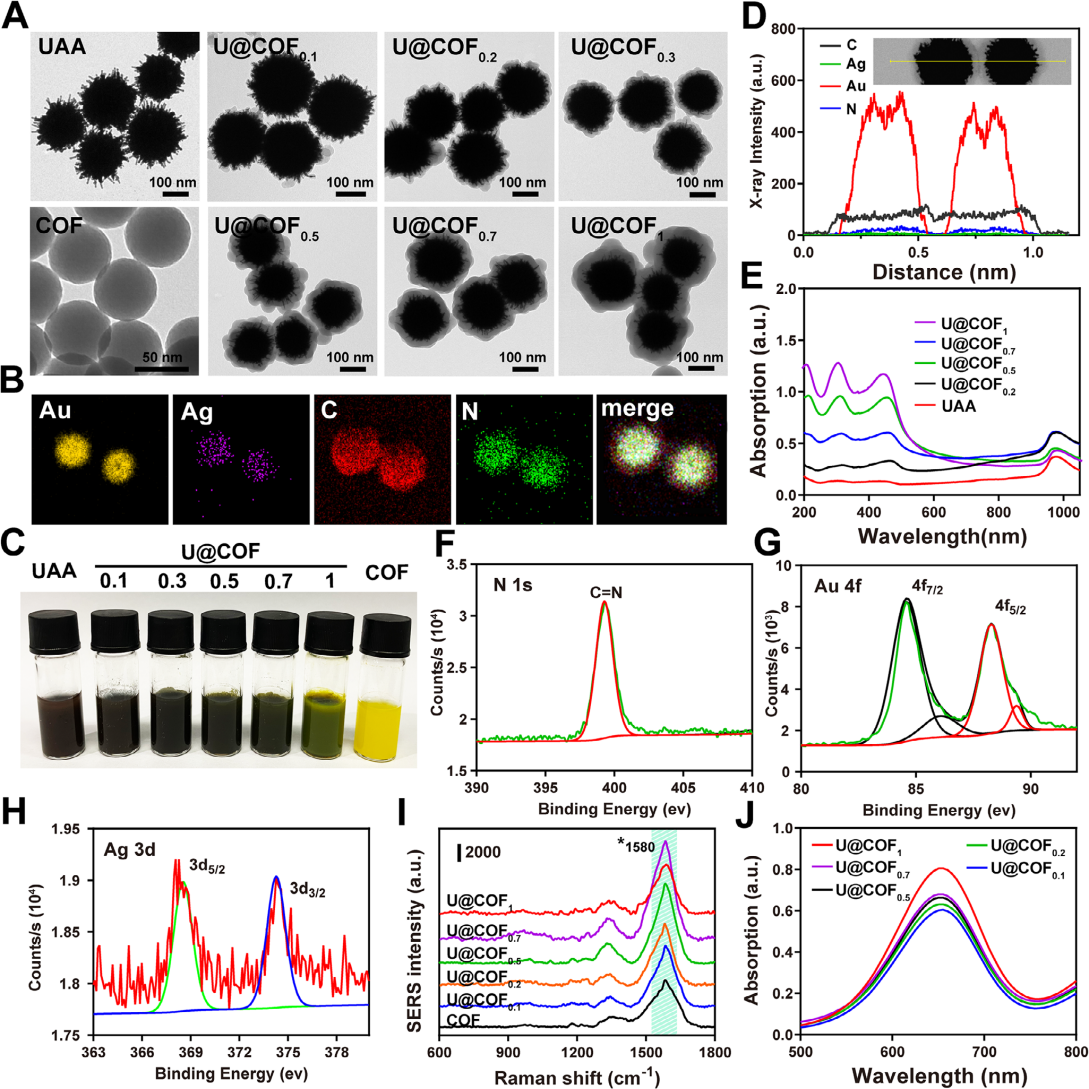
Synthesis and characterization of U@COF. (A) TEM images of UAA, COF, U@COF_0.1_, U@COF_0.2_, U@COF_0.3_, U@COF_0.5_, U@COF_0.7_, and U@COF_1_. (B) EDS elemental mapping of Au, Ag, C, and N from U@COF. (C) The color of UAA (black), COF (yellow), and U@COF synthesized with different thicknesses of COF shell (dark green) (D). The line scan of Au, Ag, C, and N from U@COF. (E) UV–vis absorption spectra of UAA and U@COF. High‐resolution (F) N 1s, (G) Au 4f, and (H) Ag 3d XPS spectra of U@COF. (I) SERS spectra of COF and U@COF. (J) UV–vis absorption spectra of TMB catalyzed by U@COF.

Transmission electron microscopy (TEM) showed that the synthesized UAA was well dispersed with a uniform size of about 200 nm (Figure 1A). Then, the growth of the COF shell enabled the transformation of morphology from urchin to flower‐like shape with unique core–shell structures, and their color changed from black to dark green (Figure 1C). A noticeable LSPR peak emerged at 450 nm after COF modification, which was mostly associated with the n‐*π* ^*^ transition of the imine group (Figure 1E). The thickness of the COF layer was modulated by adjusting the amount of precursor solution for TAPB and DMTP (0.1, 0.2, 0.3, 0.5, 0.7, and 1 mL, respectively). The thickness of the COF layer was inferred to be approximately 3, 8, 16, 25, 33, and 40 nm with the growing ratio of precursor solution. It is worth noting that without the participation of UAA, COF would spontaneously self‐polymerize into a nanosphere morphology with a significant mesoporous structure, in a uniform size of 60 nm. Besides, elemental mapping analysis in Figure 1B exhibited the chemical distribution of Au, Ag, C, and N over the whole U@COF. The line scan profile across the region in Figure 1D further demonstrated the chemical distribution of U@COF, with the highest percentage of Au elements. The TEM image further showed that U@COF had good dispersibility and uniformity from batch to batch (Figure S1). The XPS spectra displayed the C1s, N1s, and O1s peaks (Figure 1F and Figure S2), which originated from the TAPB and DMTP. From Figure 1G,H, the binding energies at 84.58 and 88.28 eV correspond to Au 4f_7/2_ and Au 4f_5/2_, and 374.28 and 368.28 eV correspond to Ag 3d_3/2_ and Ag 3d_5/2_, respectively. Furthermore, the as‐produced U@COF were further assessed by x‐ray diffraction (XRD) (Figure S3), where the diffraction peaks of U@COF at 4.8°, 5.6°, 7.4°, 9.7°, and 25.3° correspond to the (110), (200), (120), (220), and (001) crystal planes of COF, indicating the good crystallinity of COF. Moreover, a diffraction pattern at 38.2°, 44.2°, 65.6°, and 77.8° corresponded to the (111), (200), (220), and (311) lattice planes of Au/Ag, respectively, which suggested the structural integrity of U@COF. The N_2_ adsorption isotherm of U@COF was investigated. As displayed in Figure S4, U@COF presented a typical type IV isotherm inferior BET surface area (9.33 m^2^ g^−1^) and pore volume (0.04 cm^3^ g^−1^). The pore size distributions fitted by the BJH model showed large‐scale peaks, indicating its classic microporous structure, which were conducive to mass transfer and biosensing.

To shed light on the mechanism of U@COF, we evaluated several different synthetic routes and the reaction conditions to induce the assembly of U@COF. To begin, we found that the morphology of COF was closely related to the reaction solvent during polymerization, where completely different forms were obtained when dispersed in water and methanol (Figure S5). It was reasonable because solvent exchange conditions could alter the stacking mode of two monomers, causing changes in functional group orientation, molecular conformation, etc. SERS characterization further supported the optimal properties of U@COF synthesized in methanol conditions (Figure S6). When the concentration of COF precursors remains constant (DMTP 25 mm, TAPB 30 mm), the excessive amounts of UAA had a negligible effect on the formation of U@COF; conversely, insufficient amounts of UAA (0.5 or 0.8 times) would trigger the self‐aggregation of COF precursors surrounding around the U@COF (Figure S7). In addition, compared with the stirring dynamic condition, a relatively thin COF shell was formed on U@COF under static growth conditions, together with some self‐polymerized COF structures with rough morphology (Figure S8). Besides, this reaction enabled it to proceed softly and rapidly at room temperature [41]. TEM images showed that the reaction product obtained at room temperature was a complete COF shell, while those obtained with heating formed some random clusters as impurities on the COF shell (Figure S9). Moreover, reaction time is also a key factor for the formation of the COF shell [42]. The reaction was not sufficient in the system at the beginning of 2 h, where some of the UAA had not been encapsulated (Figure S10). Therefore, we extended the reaction time to 24 h to ensure a thorough reaction.

Further, the SERS activity of U@COF was investigated based on the intrinsic COF Raman properties. As illustrated in Figure 1I, a characteristic Raman band appeared at 1580 cm^−1^ after COF encapsulation, which is attributed to the crystalline honeycomb structures of COF. The performance of COF enabled SERS probes to have an identifiable signal without the modification of the Raman reporter molecule. The thickness of the COF shell has a certain influence on the SERS performance. As the COF content increased to U@COF_0.7_, the sharpest and strongest SERS signal of U@COF was obtained. The peroxidase‐mimicking activity was also investigated via a classical colorimetric assay by utilizing TMB as the colorimetric substrate. The catalytic principle of this assay was shown in Figure S11, where a colorless TMB molecule was catalyzed to blue oxidized TMB (oxTMB) in the presence of H_2_O_2_ with a significant absorption peak at 652 nm. The peroxidase‐like catalytic activity of U@COF was strong and stable even at different thicknesses of the COF layer (Figure 1J). Comprehensive consideration of the morphology, SERS, and catalytic performance, the U@COF_0.7_ was the optimal sensor for further study. The SERS signals from different batches demonstrated the high reproducibility of U@COF with a relative standard deviation (RSD) = 6.09% (Figure S12).

Moreover, the catalytic mechanism of U@COF was further estimated by recording the steady‐state kinetics (Figure S13). V_max_ of U@COF was calculated as 1.69 × 10^−8^ M/s for H_2_O_2_ substrate and 2.81 × 10^−8^ M/s for TMB, respectively. K_m_ value of U@COF against H_2_O_2_ substrate was calculated to be 0.35 × 10^−8^ m, and against TMB was 0.29 × 10^−8^ m, respectively, proving the high catalytic rate of U@COF. Furthermore, the effects of pH, temperature, and reaction time on the catalytic activity of U@COF were assessed, and the results revealed that pH = 4.0, reaction temperature of 37°C, and reaction time of 15 min exhibited the optimal enzymatic catalytic activity (Figures S14–S16). All of the results suggested the high SERS and peroxidase‐mimicking activity of U@COF, allowing it suitable for further multi‐model sensing ESAT‐6/CFP‐10 antigen.

### 3.2 Characterization of Ag@AuNF Array‐Based SERS Facemask

**FIGURE 2:**
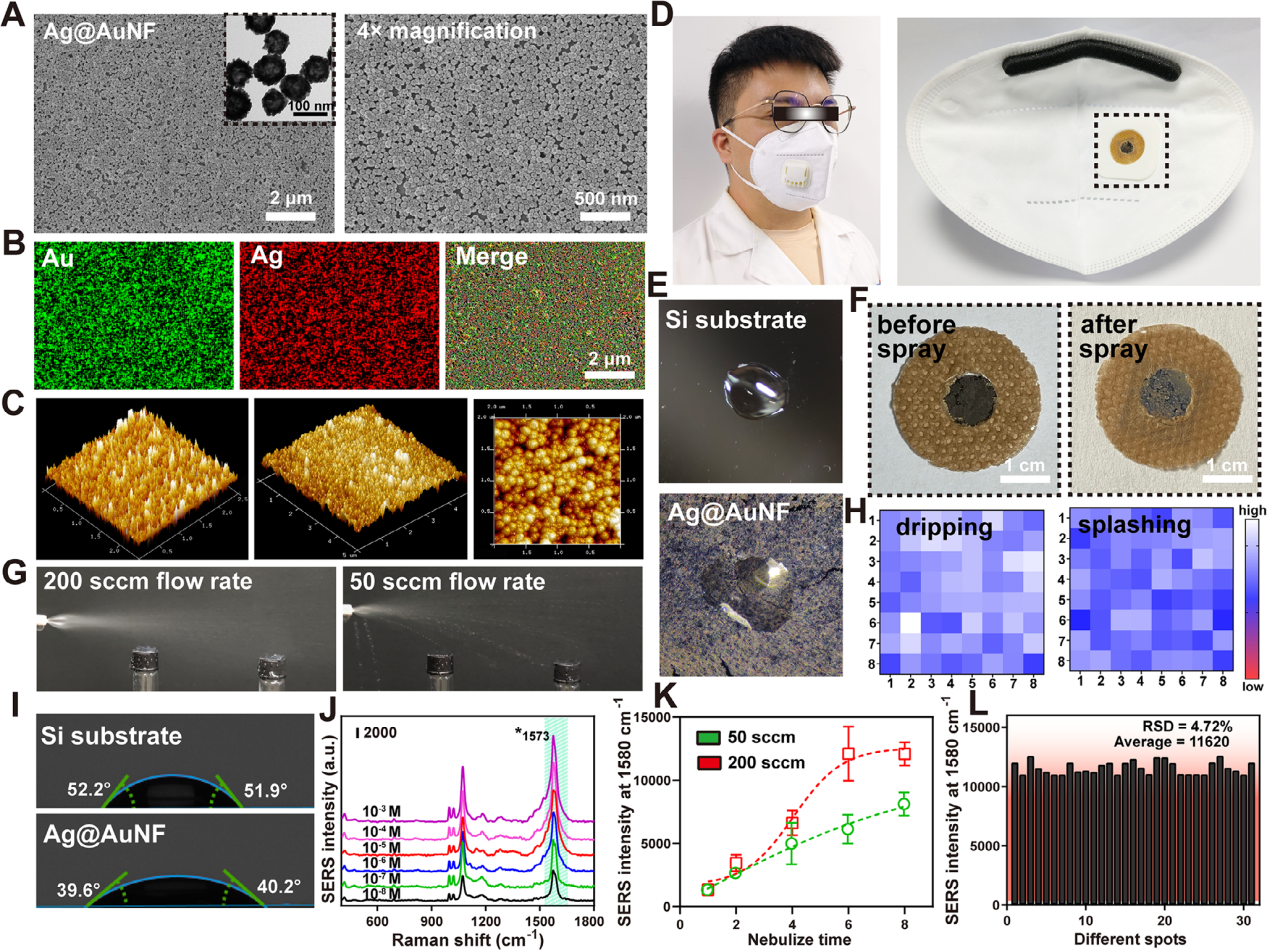
Feasibility of Ag@AuNF array‐based SERS facemask. (A) SEM image of Ag@AuNF substrate. Inset: TEM image of Ag@AuNF. (B) SEM elemental mapping images of Ag@AuNF substrate. (C) AFM image of Ag@AuNF substrate with vertical cross‐sectional profiles. (D) Optical image of a portable facemask for droplet detection. The Ag@AuNF chip was immobilized on the mask filter membrane with medical adhesive tape. (E) The diffusion area of the droplet on the Ag@AuNF chip. (F) Optical image of Ag@AuNF chip before or after splashing the droplet. (G) Spraying droplets at different speeds using a nebulizer. (H) Comparison of the SERS intensity of 4‐MPBA after dripping and splashing. (I) Contact angles of Ag@AuNF substrate. (J) Spraying different concentrations of 4‐MPBA droplets on the Ag@AuNF substrate. (K) SERS signals of nebulized 4‐MPBA at different spray speeds. Data are presented as mean ± SD. (*n = 3*). (L) SERS spectra obtained from 30 random regions of the Ag@AuNF substrate.

Here, we developed an Ag@AuNF array‐based SERS facemask for the detection of aerosolized ESAT‐6/CFP‐10 antigen. The embedded Ag@AuNF array with strong, stable, and reproducible SERS signals and high hydrophilicity enabled rapid, simple, and sensitive sensing of ESAT‐6/CFP‐10 antigen exhaled by TB patients. TEM image showed that Ag@AuNF was synthesized with a uniform morphology and good dispersibility in a high yield (>90%) (Inset in Figure 2A). Next, monodisperse Ag@AuNF at the liquid‐liquid interface was driven to self‐assemble into an ordered monolayer film via the interfacial tension, then transferred onto a solid substrate to form a dense 2D chip. As revealed by SEM (Figure 2A), regardless of the scanning area, Ag@AuNF was uniformly arranged in parallel on the array. Atomic force microscope (AFM) images showed the top view of the array, in which Ag@AuNF film was assembled by face‐to‐face contact to form a close‐packed monolayer, with heights of approximately 80 nm (Figure 2C). The uniformly distributed Ag@AuNF on the array surface was crucial for improving uniformity and repeatability in subsequent SERS analysis. The EDX elemental maps sequentially revealed a uniform distribution of Au atoms around the core area of Ag atoms, indicative of the successful fabrication of Ag@AuNF array (Figure 2B). The Ag@AuNF SERS chip was cut into small pieces and attached to the air filter of a commercial mask with medical tape (Figure 2D).

Droplets follow the principles of aerodynamics during flight and splash onto the substrate surface at high speed (2–10 m/s). Due to the low surface energy of the substrate, droplets with a large number of target analytes are bounced back upon contact with the substrate surface and cannot be effectively accumulated on it, which greatly limits the detection sensitivity. Therefore, the improvement of the substrate surface energy is of great significance for the capture of more droplet substances at the interface. The contact angle of a water droplet was then recorded to evaluate the surface energy of the Ag@AuNF chip. As presented in Figure 2I, the solid Si substrate showed a contact angle of 52.2°, while the Ag@AuNF substrate with an ordered orientation evidently decreased the contact angle to 39.6°. The high water affinity allowed analytes to condense and enrich on the chip surface for subsequent highly sensitive SERS analysis. The diffusion area of the droplet on the Ag@AuNF chip further confirmed its high surface energy (Figure 2E). Further, we converted the liquid into an aerosol through nebulization and sprayed it onto the array. The droplets were uniformly and closely deposited on the array and remained fixed on it (Figure 2F).

The SERS performance was assessed by nebulizing a Raman molecule, 4‐mercaptophenylboronic acid (4‐MPBA), onto the Ag@AuNF substrate. The nebulized 4‐MPBA directly bound and immobilized on the Ag@AuNF chip surface via Au─S bonds, exhibiting the typical Raman peaks under 633 nm excitation (Figure S17). As shown in Figure 2J, the SERS intensity gradually increased with the increase of 4‐MPBA concentration. When the 4‐MPBA concentration was higher than 10^−4^ m, the SERS intensity gradually stabilized, mainly ascribed to the saturation of array adsorption. The enhancement factor (EF) was calculated to be 2.44 × 10^8^, demonstrating the high SERS activity of the Ag@AuNF chip. The influence of droplet sputtering speed and frequency on the pre‐enriched target analytes was further investigated by measuring the nebulized 4‐MPBA signal on the array (Figure 2G). The amount of liquid deposited per unit time (3 s/time) was relatively less at a slow sputtering speed (50 sccm flow rate), the SERS signal on the array increased slowly with the increase of time (Figure 2K). On the contrary, as the splashing speed increased (200 sccm flow rate), more liquid was compressed and sprayed out. Under the same conditions of splashes, the SERS signal was stronger and reached the saturation stage more quickly when compared with the slow sputtering speed. The collection of samples is closely related to the degree and frequency of cough in patients. In addition, we compared the differences between direct dripping and splashing droplets on the array. The SERS mapping showed similar SERS signals between the two methods, further confirming the feasibility of SERS‐facemask for droplet detection (Figure 2H). These explorations provided a reference for the further investigation of the clinical application of the SERS‐facemask.

To estimate the reproducibility of this strategy, the SERS spectra of 4‐MPBA were randomly collected on 30 spots on the Ag@AuNF substrates of the facemask. As depicted in Figure 2L, the spectra presented a similar band without a significant change in the SERS signal intensity. The detection repeatability was calculated based on the intensity of the characteristic peaks at 1573 cm^−1^, where the low RSD value (RSD = 4.72%) revealed the superior reproducibility.

### 3.3 Feasibility of ESAT‐6/CFP‐10 Detection

**FIGURE 3:**
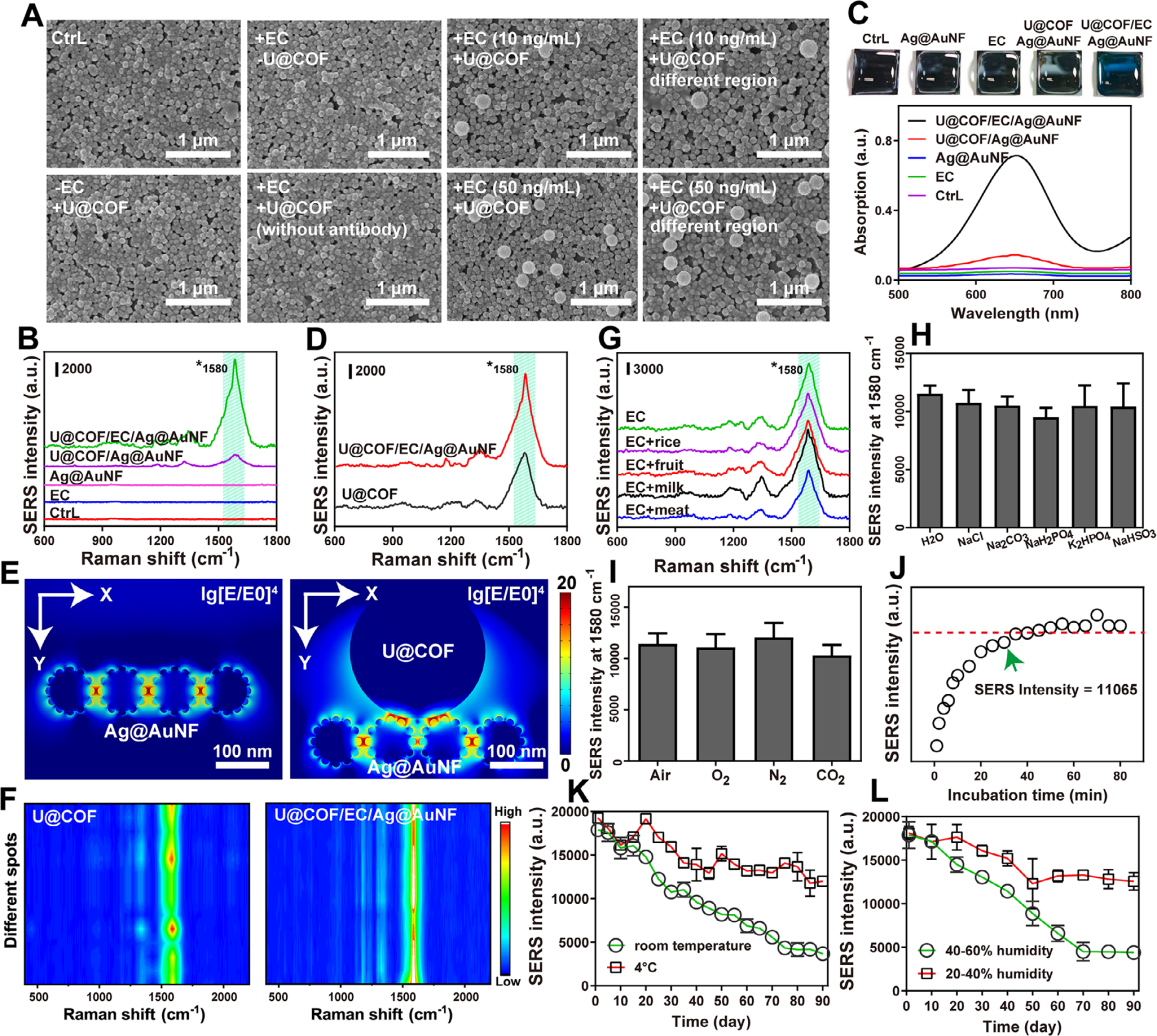
Feasibility of ESAT‐6/CFP‐10 detection. (A) SEM image, (B) TMB catalysis, and (C) SERS spectra revealed the availability of this nanosatellite strategy. (D) SERS performance of U@COF or U@COF‐mediated satellite complexes. (E) FDTD simulation depicting the electromagnetic near‐field distribution for Ag@AuNF array before or after U@COF complexation. (F) 2D Raman spectra for (D) (*n = 60*). (G) SERS detection of ESAT‐6/CFP‐10 in the presence of interfering substances (rice, fruit, milk, and meat). (H) SERS detection of ESAT‐6/CFP‐10 under different condition (H_2_O, NaCl, Na_2_CO_3_, NaH_2_PCO_4_, K_2_HPO_4_, and NaHSO_3_). (I) SERS detection of ESAT‐6/CFP‐10 under different conditions (air environment, O_2_, N_2_, and CO_2_). (J) SERS intensities (I_1580_ cm^−1^) of ESAT‐6/CFP‐10 at different incubation times. (K) SERS performance of Ag@AuNF array stored at 4°C and room temperature. (L) SERS performance of Ag@AuNF array stored at 20%–40% and 40%–60% humidity. Data are presented as mean ± SD. (*n = 3*).

Herein, the satellite nanoassemblies were constructed based on aptamer‐decorated Ag@AuNF substrate (capture substrate) and antibody‐modified U@COF (catalytic/SERS sensing probe) for detecting ESAT‐6/CFP‐10 (EC). The aptamer‐antibody competition binding was evaluated, and the results are shown in Figure S18, where antibodies did not affect the binding of aptamer to EC heterodimers, indicating that the binding sites of aptamer and antibody do not overlap. The two recognition modes can effectively avoid the mutual interference between recognition sites and improve recognition efficiency. Figure S19 further revealed that CFP‐10 antibodies could effectively bind to the CFP10/ESAT6 heterodimers, due to the spontaneous formation of CFP10/ESAT6 heterodimers under physiological conditions. SEM images clearly showed that in the absence of target EC, there was only a uniform layer of Ag@AuNF chip, similar to the blank control (Figure 3A). Furthermore, U@COF without antibody decoration could not capture target EC and crosslink with Ag@AuNF array well. When the target EC was introduced, antibody‐modified U@COF with an obviously large size was evenly deposited on the surface of Ag@AuNF array, and the accumulation of U@COF increased with the rise of EC concentration. Interestingly, irrespective of which position was scanned for observation, U@COF was uniformly distributed on the Ag@AuNF array (Figure S20), which was conducive to the uniform and stable output of the subsequent SERS signal.

Leveraging the intrinsic Raman characteristics of U@COF, the feasibility was assessed by setting several controlled experiments. As presented in Figure 3C, in the absence of EC, U@COF could almost not attach to the Ag@AuNF substrate. Only when the EC antigen to induce the conjugation of U@COF/Ag@AuNF substrate can the distinct SERS signal be observed. Moreover, the SERS response was further verified by a classical colorimetric assay due to the peroxidase‐mimicking activity of U@COF. The results in Figure 3B showed that when the EC target was captured on the Ag@AuNF array, U@COF catalyzed colorless TMB substrate into blue oxTMB gradually, which further validated the successful detection of target EC by U@COF‐mediated satellite nanostructure.

U@COF has significant Raman enhancement properties, and the integration of the Ag@AuNF array further amplified the SERS intensity due to the formation of the dual‐plasmonic satellite nanoassemblies. To validate the dual‐SERS enhancement effect, we compared the SERS performance of U@COF before or after conjugation with Ag@AuNF array. As a result, the SERS signal was amplified due to the aggregated “hot spots” between adjacent U@COF and Ag@AuNF (Figure 3D). To deeply understand the electric field enhancement effect, finite‐difference time‐domain (FDTD) analysis was conducted. The simulation results indicated that compared with Ag@AuNF, the plasmonic coupling between Ag@AuNF and U@COF induced a gap‐mode resonance, generating a highly enhanced electromagnetic field (Figure 3E). To further assess signal strength and uniformity, a total of 60 spots were randomly acquired on the U@COF substrate before or after incubation with the Ag@AuNF array. According to the 2D SERS image (Figure 3F), SERS enhancement of nanoassemblies was much stronger than that of the U@COF substrate. Additionally, the similar SERS results in different regions of the same chip or multiple chips confirmed the favorable reliability of this nanosatellite strategy (Figure S21).

Considering that the composition of the daily diet might affect the SERS detection, common food items, such as rice, meat, milk, and fruits, were extracted, mixed with EC solution, and splashed onto the SERS substrate. Results in Figure 3G illustrated that despite the addition of different components, the SERS signals remained almost unchanged, which indicated that the interaction of different molecules in the mixture did not interfere with the SERS detection. In addition, the interference of the inorganic salts on the testing was further investigated. Obviously, the results showed the influence of these components on the SERS detection was negligible (Figure 3H). Moreover, the impact of the detection environment was also evaluated by exposing it to various interfering conditions, including oxygen gas (O_2_), nitrogen (N_2_), carbon dioxide (CO_2_), and air environment. As shown in Figure 3I, SERS signals showed no significant differences under different conditions, confirming the robust stability of this method. The incubation time was analyzed, and the results revealed that reaction time must be longer than 30 min, within which the interaction of satellite nanoassemblies was completed, and the SERS signal tended to stabilize (Figure 3J). However, the SERS signal of Ag@AuNF array showed a decline after being stored for 90 days (Figure 3K). The instability might be caused by the easy oxidation of Ag NP, where the morphology of Ag NP has shown obvious collapse and deformation (Figure S22). It is worth noting that low temperature storage (4°C) can delay the change of the properties of the Ag@AuNF array. Additionally, the storage environment of Ag@AuNF array should be kept dry, as a humid environment has a significant impact on its SERS detection performance (Figure 3L). Therefore, we recommend using moisture‐proof beads or placing in a moisture‐proof box to control the storage humidity and extend the service life of SERS masks.

### 3.4 Performance of Facemask Sensor for ESAT‐6/CFP‐10 Detection

**FIGURE 4:**
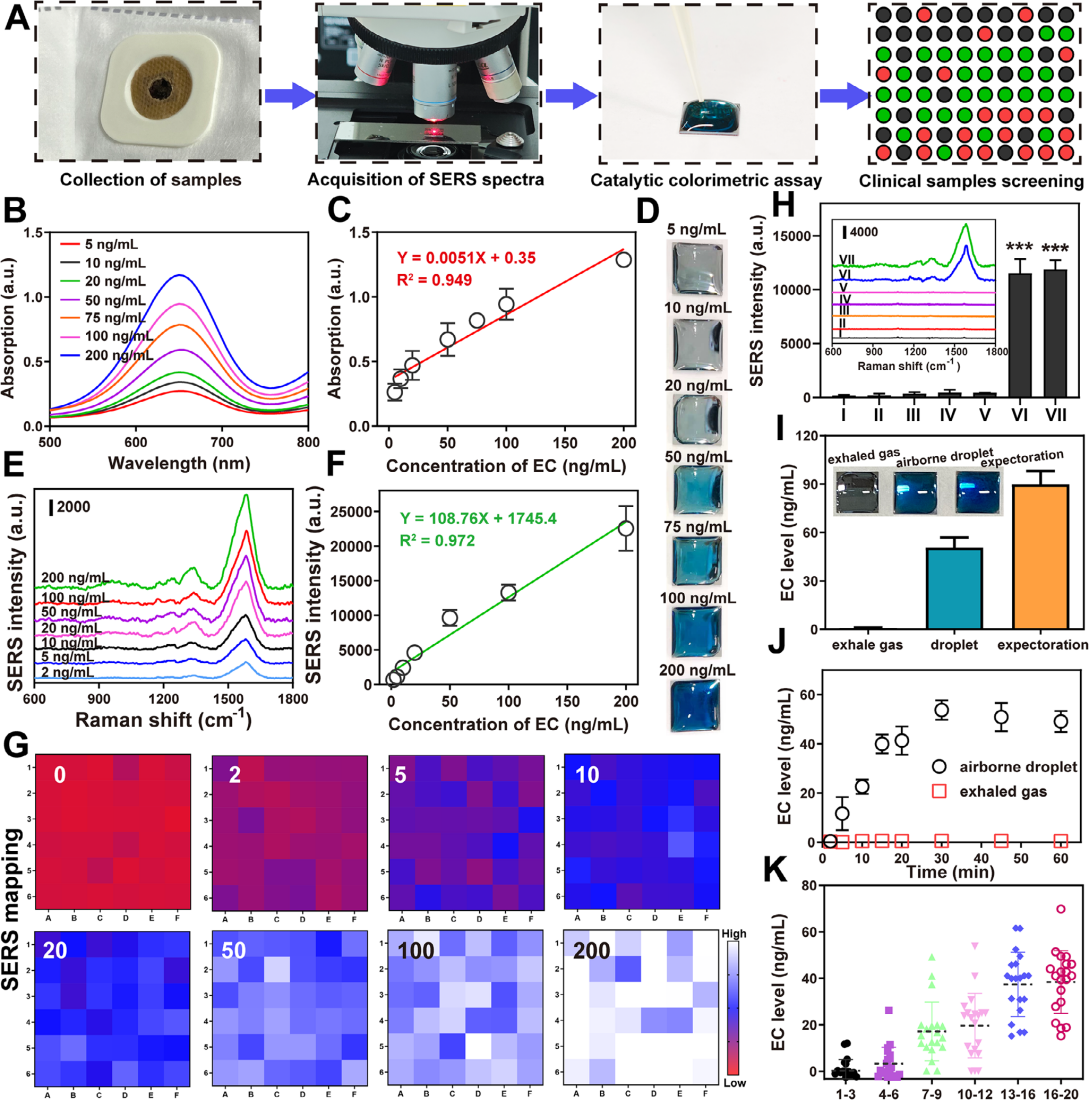
Performance of mask device for ESAT‐6/CFP‐10 detection. (A) The detection process of the SERS‐facemask. (B) Catalytic colorimetric assay in various concentrations of ESAT‐6/CFP‐10. (C) Linear relationships between ESAT‐6/CFP‐10 concentration and absorption intensity at 652 nm (5–200 ng/mL). (D) Photograph of TMB solutions treated as in (B). (E) SERS assay in various concentrations of ESAT‐6/CFP‐10. (F) Linear relationships between ESAT‐6/CFP‐10 concentration and SERS intensity at 1580 cm^−1^ (2–200 ng/mL). (G) SERS mapping images (6 × 6 µm^2^) taken at 1580 cm^−1^ with the increasing concentration of ESAT‐6/CFP‐10 (ng/mL). (H) Specificity analysis of the facemask test. Inset: SERS spectra for each group. (I to VI = Ag85B, AFP, PSA, LAM, LPS, and ESAT‐6/CFP‐10; VII = Mixture + ESAT‐6/CFP‐10). ^**^, *p* < 0.01; ^***^, *p* < 0.001. (I) ESAT‐6/CFP‐10 levels in exhaled gas, airborne droplets, and expectoration sample. Inset: the photograph of blue oxTMB in different groups. (J) ESAT‐6/CFP‐10 levels in exhaled gas and droplet samples at different wearing times. Data are presented as mean ± SD. (*n = 3*). (K) ESAT‐6/CFP‐10 levels in air droplet samples at different coughing times.

Considering the airborne droplet or expectorations contained substantial TB‐specific markers [43, 44], we herein presented a proof‐of‐concept application where Ag@AuNF substrate was integrated into the mask, which can automatically capture ESAT‐6/CFP‐10 from airborne droplets or expectorations, and removed after wearing for a certain period (Figure 4A). Benefiting from the hydrophilic ability of Ag@AuNF substrate, ESAT‐6/CFP‐10 samples were collected and adsorbed on the chip, followed by incubation with U@COF for SERS/colorimetric detection. This mask was developed for TB patients with cough, and the effective arrangement of chips can meet the requirements for screening a large number of clinical samples.

During the experiment, ESAT‐6/CFP‐10 were dissolved in PBS, filled into the jetting device, and sputtered onto the array in an aerosol state under high pressure to further evaluate the feasibility and sensitivity of the assay. After natural air‐drying, U@COF was added to conjugate with the target ESAT‐6/CFP‐10 and catalyze the oxidation of TMB to a blue oxTMB solution for rapid visualization. As revealed in Figure 4B,D, the colorimetric and absorption intensity of oxTMB intensified substantially with the increase of ESAT‐6/CFP‐10 concentration. The linear regression equation was depicted as Y = 0.0051X + 0.35 within the range of 5–200 ng/mL (R^2^ = 0.949), where Y represented the absorbance intensity at 652 nm; X represented the target concentration (Figure 4C). For SERS analysis, the feature peak of U@COF was still detectable even when the concentration was reduced to 2 ng/mL (Figure 4E), verifying the high sensitivity of SERS sensing. The great calibration correlation between the target concentration and the SERS intensity at 1580 cm^−1^ was validated, where the calibration curve was calculated as Y = 108.76X + 1745.4 (R^2^ = 0.972) with LOD of 0.437 ng/mL (Figure 4F). Table S1 compared the previous advanced methods for CFP‐10/ESAT‐6 analysis, which further highlighted the superiority of our platform. More importantly, the as‐developed mask can quickly and sensitively detect the CFP‐10/ESAT‐6 in droplets or sputum, providing a new portable method for TB diagnosis. SERS/colorimetric dual‐mode sensing can meet the detection requirements of visualization and sensitivity, and mutually verify the measurement results, thereby reducing the false positive/negative. The SERS mapped images were scanned over an area of 6 × 6 µm^2^ (Figure 4G), where uniform output at different detected zones demonstrated the homogeneous and dense “hot spots” of the 2D substrate, and the increased linear signal indicated the gradual accumulation of U@COF immunocomplexes.

To further evaluate the specificity of the proposed system, a series of interfering substances (MTB antigen Ag85B, alpha fetoprotein (AFP), lipoarabinomannan (LAM), prostate specific antigen (PSA), and Lipopolysaccharide (LPS), 500 ng/mL) were included as typical controls. It was clear that only the target ESAT‐6/CFP‐10 displayed an obvious signal, whereas other interfering substances showed a weak signal similar to the blank group (Figure 4H). Moreover, the mixture of the above interferents presented a negligible impact on the original signal of ESAT‐6/CFP‐10, all of which confirmed the high specificity and anti‐interference performance of the proposed system. It should be attributed to the U@COF and Ag@AuNF dual‐recognition system, where only the target can be effectively captured, even in a mixed complex detection. Furthermore, the aggregated “hot spots” between adjacent U@COF and Ag@AuNF substrate evidently intensified SERS enhancement, vastly improving the sensitivity of the assay. Therefore, these satisfactory specificity, repeatability, and stability make it promising for further application in actual samples.

To investigate whether the sputum components would interfere with the analysis, recovery experiments were conducted by spiking different concentrations of ESAT‐6/CFP‐10 (20, 50, and 100 ng/mL) into sputum samples. The results in Table S2 showed that the immunoassay has a good recovery within the range of 89.5%–106.5%, confirming the practical feasibility of this method for detecting ESAT‐6/CFP‐10 in clinical samples. To assess the clinical performance of this method, TB patients were recruited by the Ninth People's Hospital of Dongguan (Dongguan Infectious Disease Hospital), and exhaled gas, airborne droplets, and expectoration were collected by wearing an SERS‐facemask for analysis. As depicted in Figure 4I, cases showed almost undetectable values of ESAT‐6/CFP‐10 in exhaled gas. We thus prolonged the wearing time of the patient to ensure full contact between the exhaled substances and the sensing substrate. Nevertheless, the signal of the target ESAT‐6/CFP‐10 was still exceedingly low (Figure 4J), even when the same experiment was conducted on another patient (Figure S23). Therefore, we further investigated the feasibility of this mask sensor in air droplets or expectoration samples. As we expected, air droplets coughed up from the lungs showed a significant ESAT‐6/CFP‐10 signal, and the signal was further increased in the expectoration samples, which is reasonable because the rapid airflow of coughing will carry away some of the target substances, and the sample amount of air droplets is much smaller than that of expectoration. The SERS results were further supported by the catalytic colorimetric assay, where the blue oxTMB was visualized obviously in the airborne droplet or expectoration sample (inset images in Figure 4I). Furthermore, as the duration for which patients wore masks varied, more airborne droplets were excreted through coughing, and the signal of ESAT‐6/CFP‐10 also increased accordingly (Figure 4J). The signal value reached saturation within 30 min, indicating that the detection procedure is completed. However, considering the sampling process takes a long time, which is not suitable for rapid screening or on‐site detection, we shortened the wearing time of patients to 15 min, at which the collected signal has exceeded 80% of the plateau period, making it available for clinical scenarios.

Similar to the extension of wearing time, the times of coughing have a significant influence on ESAT‐6/CFP‐10 testing. The results showed that with the increase in the coughing times, the amount of air droplets secreted during coughing also gradually increased (Figure 4K). When coughing remained between 13 and 16 times, the signal tended to stabilize. It is worth noting that the wearing time and cough times only provided a general reference range, as the content of ESAT‐6/CFP‐10 is also related to the severity of the patient's symptoms. It might be possible that the time for detection will be shorter if the symptoms are more severe.

### 3.5 Clinical Samples Analysis of TB

**FIGURE 5:**
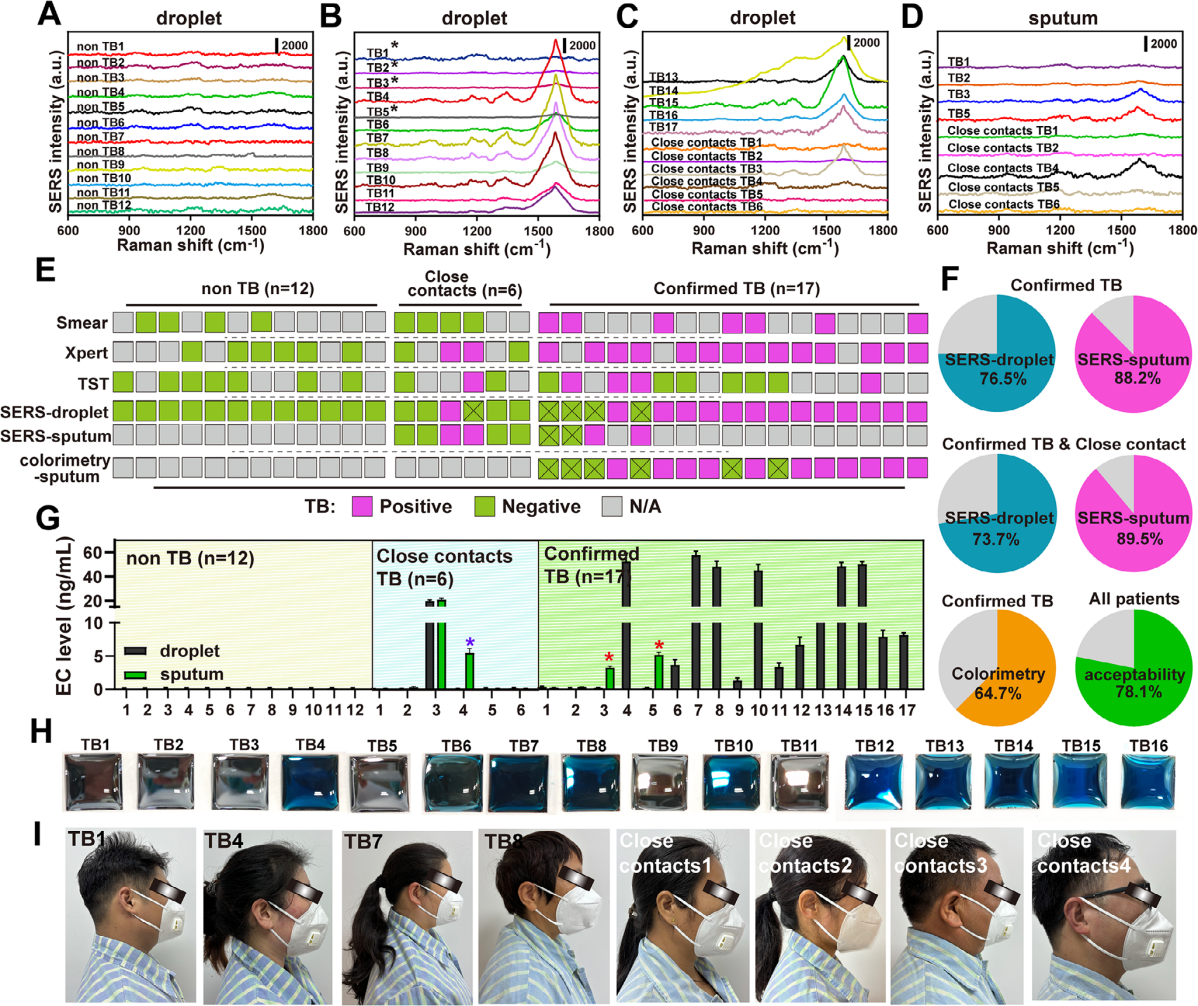
Clinical samples analysis of TB patients. (A–C) SERS‐facemask detection of droplet samples from different subjects. (D) SERS‐facemask detection of sputum samples from different subjects. (E) Cluster map of clinical and facemask test results for all samples. Clinical tests include smear, Xpert, and TST. Pink block and green block indicate the positive and negative of TB infection, respectively. *grey block* indicates not applicable, × indicates the incorrect result of our detection. (F) Sensitivity and acceptability of TB detection. (G) Quantification of ESAT‐6/CFP‐10 levels in different samples by SERS‐facemask. Data are presented as mean ± SD. (*n = 3*). (H) Colorimetric visualization for quickly identifying TB infection. (I) Different patients wearing masks.

Compared with traditional microbiological testing, such as sputum smear microscopy and culture test, direct detection of circulating ESAT‐6/CFP‐10 antigens in air droplets or sputum through a mask device can avoid problems such as complicated sampling, viable bacterial cells test, time‐consuming and labor‐intensive, and is particularly suitable for application in high‐risk places or areas with limited resources. More importantly, wearing masks is an effective way to cut off the transmission routes of TB, thus the SERS mask designed in this study is a comprehensive system that combines protection and detection. To evaluate the clinical application value of the mask device for TB diagnosis, total 35 participants were recruited and retrospectively categorized into “non TB” (*n = 12*), “confirmed TB” (*n = 17*), and “close contacts (*n = 6*),” according to the clinical discrimination. Researchers conducted double‐blind tests and compared the obtained SERS/colorimetric data for further analysis. The SERS signal (I_1580_) of each sample was measured and converted to ESAT‐6/CFP‐10 level by the standard curves. The positive (pink patches) and negative (green patches) samples determined from SERS and clinical tests (smears, Xpert, and TST) were shown in Figure 5E, where the grey patches indicated the invalid information provided by the hospital.

The ROC analysis was performed to obtain the diagnostic threshold value of 0.7485 ng/mL (Figure S24), from which the result was determined as positive (above the threshold) or negative (below the threshold). The optimal sensitivity and specificity values provided by the ROC analysis were 76.5% and 100%, respectively. Figure 5A showed that all 12 samples of non‐TB infections were successfully recognized. Among the 17 samples with confirmed TB (Figure 5B,C), four (TB1, TB2, TB3, and TB5, denoted by black stars) were designated as negative by the SERS assay, which was inconsistent with the results from clinical testing. However, they presented positive sputum smear results from the initial screening, or further confirmed TB positivity by later Xpert analysis. Thus, these four data measured by SERS were deemed as false negatives, possibly because the content of ESAT‐6/CFP‐10 antigen complex in the droplets spat out during coughing falls below the detection threshold of SERS. Considering the abundant ESAT‐6/CFP‐10 distributed in the sputum samples, we re‐collected the sputum samples of these four patients for SERS testing (Figure 5D). As expected, two TB‐infected patients’ sputum (TB3 and TB5, marked by red stars) were re‐identified as a positive sample (Figure 5G), and the sensitivity of SERS sensing was increased from the original 76.5% (13/17) to 88.2% (15/17) (Figure 5F).

Due to the relatively low sensitivity of catalytic colorimetry compared to SERS sensing, six positive samples were missed by the colorimetric assay (Figure 5E,H), with a sensitivity of merely 64.7% (11/17) (Figure 5F). The two false‐negative samples (TB9 and TB11) were also negative in the TST test, but positive in the Xpert or smear tests (Figure 5E). However, the colorimetric method has the characteristics of simplicity, convenience, and visualization, and thus is suitable for the rapid screening of severe patients in the early stage of infection. Therefore, this colorimetric/SERS dual‐mode sensing can meet the detection requirements at different levels and offer a mutual check of the results.

This portable mask sensor held the potential to screen the TB close contacts. Close contact with TB patients were enrolled for further TB assessment (*n = 6*), regardless of whether they had pulmonary TB symptoms. The SERS results showed that one TB patient was screened out (Close contact 3) in the droplet samples, who was a family member of a TB‐infected patient (TB4) (Figure 5C,I). Furthermore, their sputum sample was also collected for further confirmation, and the result was consistent with the droplet samples, showing the obvious SERS signal. However, in the sputum samples, another sample (Close contact 4, marked by a purple star) was identified as positive, which had been missed in the previous droplet test with inconspicuous symptoms of TB infection (Figure 5E). To further determine whether this patient (Close contact 4) was infected, we reviewed the clinical records and confirmed that this patient was positive on both the early TST and later Xpert test. Combining the samples of close contact, only two patients confirmed positive, we recalculated the diagnostic sensitivity of the SERS assay within this cohort (confirmed TB‐plus‐close contact:17 + 2 = 19). The sensitivity (89.5%, 17/19) for the sputum test was significantly higher than that of the droplet test (73.7%, 14/19) (Figure 5F). However, droplet samples are not as viscous as sputum; they can be directly detected without pretreatment, which is more convenient. Considering sputum sample testing is also a non‐destructive analysis with high patient compliance [45], sputum samples can be used as an auxiliary diagnosis for further improving the accuracy and reliability of droplet detection. It is worth noting that only a few patients whose data are inconsistent with those provided clinically need to undergo sputum re‐examination.

In the feedback from patients, over 75% of patients believed that compared with traditional invasive testing methods, SERS‐facemask detection offered a more comfortable wearing experience (Figure 5I). Moreover, due to its portable and wearable advantages, it can be used to screen close contacts of TB infection on a large scale. The original data and details of these clinical samples, including age, gender, infection information, and ESAT‐6/CFP‐10 levels, were exhibited in Tables S3–S5. All these encouraging results provide an attractive avenue for early diagnosis of TB, which can not only effectively distinguish infected patients from non‐infected patients, but also be used for TB screening, especially for close contacts of TB patients.

## 4 Conclusion

To conclude, we herein developed an Ag@AuNF array‐based sensing facemask, and conjugated it with catalytic/plasmonic U@COF to form plasmonic satellite nanocomplexes, which can detect the ESAT‐6/CFP‐10 biomarker in clinical droplets or sputum for the diagnosis of TB patients and the screening of close contacts of TB. Compared to the “gold standard” of MTB culture/TST/Xpert method, this work offered a noninvasive and portable strategy for TB diagnosis with high patient compliance and strong anti‐interference capacity. This SERS/colorimetric dual‐mode strategy boosted analysis reliability and sensitivity. Based on these favorable performances, the clinical applicability of this portable mask sensor was examined in clinical samples from healthy individuals and TB patients. Additionally, two close contacts of the TB patient (Close contact 3 and 4) were successfully screened out by this mask system, which corresponded well with clinical reference information. Such highly sensitive and selective mask sensors might be easily extended to other POCT fields for early disease monitoring and screening. However, considering that not all TB patients and close contacts were accompanied by coughing symptoms, this mask is not suitable for the diagnosis of asymptomatic carriers. Future research will focus on developing new systems to meet the requirements of patients without cough or those with difficulty in expectoration.

## Author Contributions

L.C., J.Y., J.Z., X.H., and H.Z. designed and supervised the project. J.X., L.C., N.Z., W.L., R.Z., X.L., Y.C., J.L., W.H., X.L., T.Z., S.C., H.C., P.S., and J.P. collected experimental samples and performed the experiments. W.L. and Y.C. recruited the clinical cohort. L.C., J.Y., X.H., and H.Z. analyzed the experimental data. X.H. and H.Z. wrote the manuscript. All authors discussed the results of this manuscript.

## Conflicts of Interest

The authors declare no conflicts of interest.

## Supporting information




**Supporting File**: advs76014‐sup‐0001‐SuppMat.docx.

## Data Availability

The data that support the findings of this study are available from the corresponding author upon reasonable request.
