# Supplementary material for: SERS Facemask for Rapid and Portable Sensing Mycobacterium Tuberculosis Antigens for TB Screening
Source: Adv Sci (Weinh). 2026 Jun 19:e23921. Online ahead of print. doi: 10.1002/advs.202523921 (PMC13336378; doi:10.1002/advs.202523921)
Supplement: Supplementary file 1 — Supporting File: advs76014‐sup‐0001‐SuppMat.docx. [file ADVS-9999-e23921-s001.docx]

**Supporting Information**

**SERS facemask for rapid and portable sensing Mycobacterium tuberculosis antigens for TB screening**

Lingzhi Chen ^a, 1^, Jiaqi Yu ^b, 1^, Jing Xu ^a^, Lingqi Chen ^b^, Ninghao Zhang ^b^, Wenrui Li ^e^, Runmin Zeng ^a^, Xiaomin Luo ^b^, Yiqun Chang ^a^, Jiayin Lai ^a^, Wenshi Huang ^a^, Xiaochen Liang ^a^, Ting Zhao ^a^, Shanze Chen ^a^, Huaihong Cai ^f^, Yanguang Cong^b^, Pinghua Sun ^c^, Jiang Pi ^b,*^, Xueqin Huang ^b, *^, Haibo Zhou ^a,b, c, *^ Junxia Zheng ^d, *^

^a^ State Key Laboratory of Bioactive Molecules and Druggability Assessment, Guangdong Basic Research Center of Excellence for Natural Bioactive Molecules and Discovery of Innovative Drugs, The Fifth Affiliated Hospital, College of Pharmacy, Jinan University, Guangzhou 510632, China.

^b^ The First Dongguan Affiliated Hospital, Guangdong Provincial Key Laboratory of Medical Immunology and Molecular Diagnostics, School of Medical Technology, Guangdong Medical University, Dongguan 523000, China.

^c^ Institute for Safflower Industry Research, Key Laboratory of Xinjiang Phytomedicine Resource and Utilization, Ministry of Education, School of Pharmacy, Shihezi University, Shihezi 832003, China.

^d^ School of Biomedical and Pharmaceutical Sciences, Guangdong University of Technology, Guangzhou 510006, China

^e^ The Ninth People’s Hospital of Dongguan, Dongguan 523000, China.

^f^ College of Chemistry and Materials Science, Jinan University, Guangzhou 510632, China

^1^These authors contributed equally to this work

* Corresponding authors: xqhuang@gdmu.edu.cn (Xueqin Huang), jiangpi@gdmu.edu.cn (Jiang Pi), haibo.zhou@jnu.edu.cn (Haibo Zhou), junxiazheng@gdut.edu.cn (Junxia Zheng).

**Table of Contents**

[Calculation of enhancement factor 3](#_Toc91771996)

[Figure S1 4](#_Toc91771996)

[Figure S2](#_Toc91771997) 4

[Figure S3 4](#_Toc91771998)

[Figure S4 5](#_Toc91771999)

[Figure S5 5](#_Toc91772000)

[Figure S6 5](#_Toc91772001)

[Figure S7](#_Toc91772002) 6

[Figure S8 6](#_Toc91772003)

[Figure S9 6](#_Toc91772004)

[Figure S10 7](#_Toc91772005)

[Figure S11 7](#_Toc91772006)

[Figure S12 7](#_Toc91772004)

[Figure S13 8](#_Toc91772005)

[Figure S14 9](#_Toc91772006)

[Figure S15 9](#_Toc91772004)

[Figure S16 10](#_Toc91772005)

[Figure S17 10](#_Toc91772005)

[Figure S18 11](#_Toc91772006)

[Figure S19 12](#_Toc91772004)

[Figure S20 12](#_Toc91772005)

[Figure S21 13](#_Toc91772005)

[Figure S22 13](#_Toc91772004)

[Figure S23 14](#_Toc91772005)

[Figure S24 14](#_Toc91772005)

[Table S1 14](#_Toc91772006)

[Table S2 15](#_Toc91772006)

[Table S3 15](#_Toc91772006)

[Table S4 16](#_Toc91772006)

[Table S5 17](#_Toc91772006)

*Calculation of enhancement factor*

The enhancement factor (EF) was calculated according to this equation

$$EF=\frac{I_{SERS}\times N_{bulk}}{I_{bulk}\times N_{SERS}}$$

where *I_bulk_* and *I_SERS_* are the intensity of bulk Raman spectra and SERS of analytes in solution, respectively. *N_bulk_* and *N_SERS_* indicate the number of molecules in the spots excited by the laser spots in bulk Raman spectra and SERS, respectively.

$$N_{SERS}=N_{A}\times CV\frac{S_{Laser}}{S_{sub}}$$

*N_A_* represent Avogadro constant; C and V correspond to the molar concentration and volume, respectively; *S_Laser_* and *S_Sub_* are the size of the laser spot and substrate, respectively. Therefore, 4-MPBA is dispersed on a clean Si substrate at V_SERS_ volume and C_SERS_ concentration.

$$N_{bulk}=N_{A}\times P_{v}S_{Laser}$$

*Pv* [mol/μm^3^] represents the volume density of 4-MPBA powder on a glass slide. During this calculation process, mass density of 4-MPBA powder is 1.27 g/cm^3^, while molecular weight of 4-MPBA is 153.1 g/mol, thus it can be calculated as *Pv* [mol/μm^3^] = (1.27/153.1) × 10^-12^ = 8.30 ×10^-15^ mol/μm^3^.

$$EF=\frac{I_{SERS}\times P_{v}S_{sub}}{I_{bulk}\times CV}$$

In this experiment, 20 μL of 4-MPBA (1×10^-9^ M) was incubated with Ag@AuNF substrate, and dried in the air to form a circle with a diameter of 4368 μm. The Raman signal of 4-MPBA was analyzed, where *I_SERS_* (8368) was significantly stronger than that of *I_bulk_* (852) at the characteristic peak of 1573 cm^-1^. Therefore, EF can be calculated as: EF = (8368 counts×8.30×10^-15^ mol/μm^3^×(4368 μm)^2^×3.14)/(852 counts×1.0×10^-9^ M×20 μL×10^-6^) =2.44×10^8^.

*Supplementary Figures and Table*


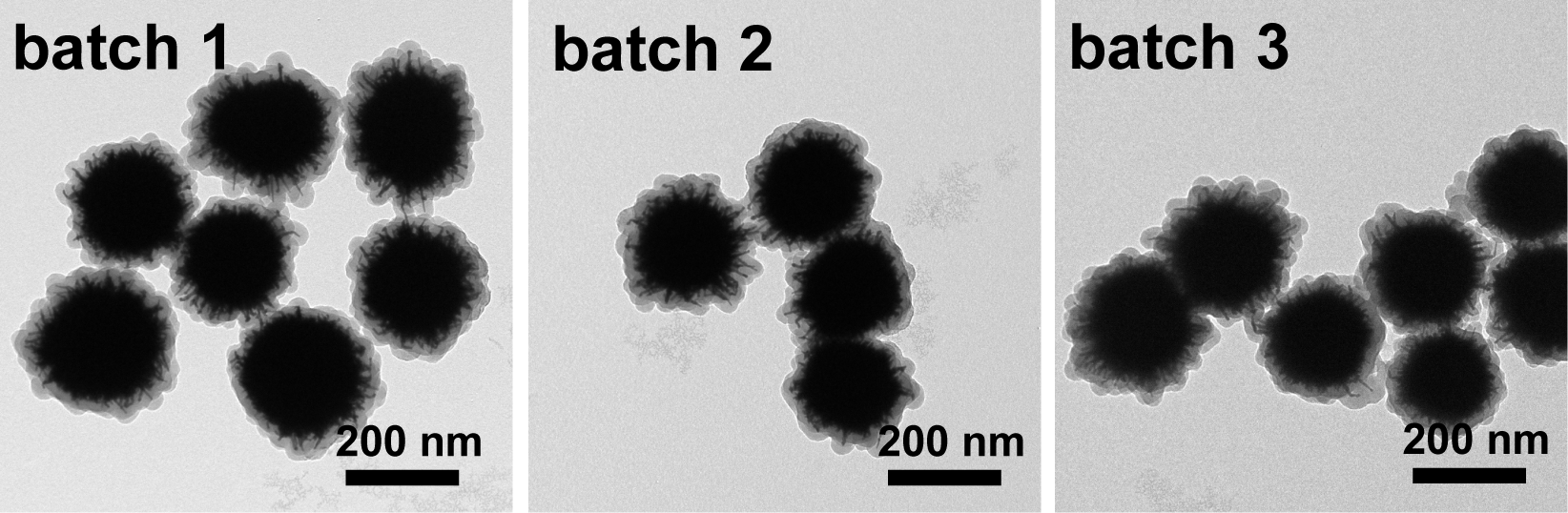


Figure S1. TEM image of U@COF in different batches.


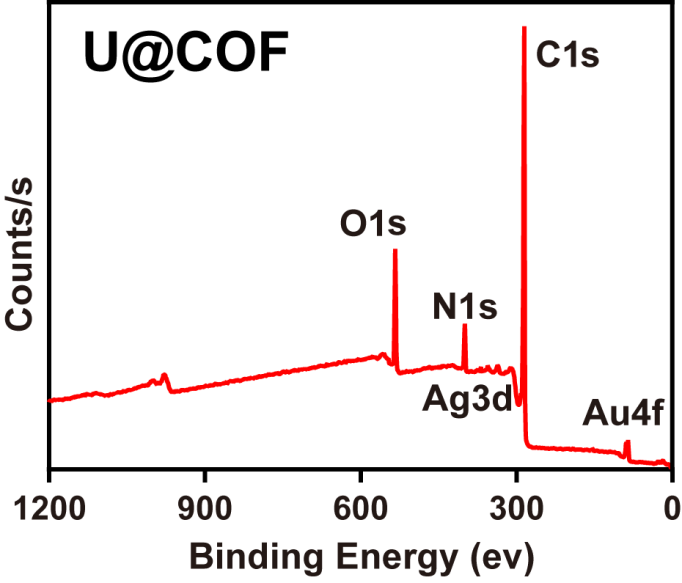


Figure S2. XPS survey scan of U@COF.


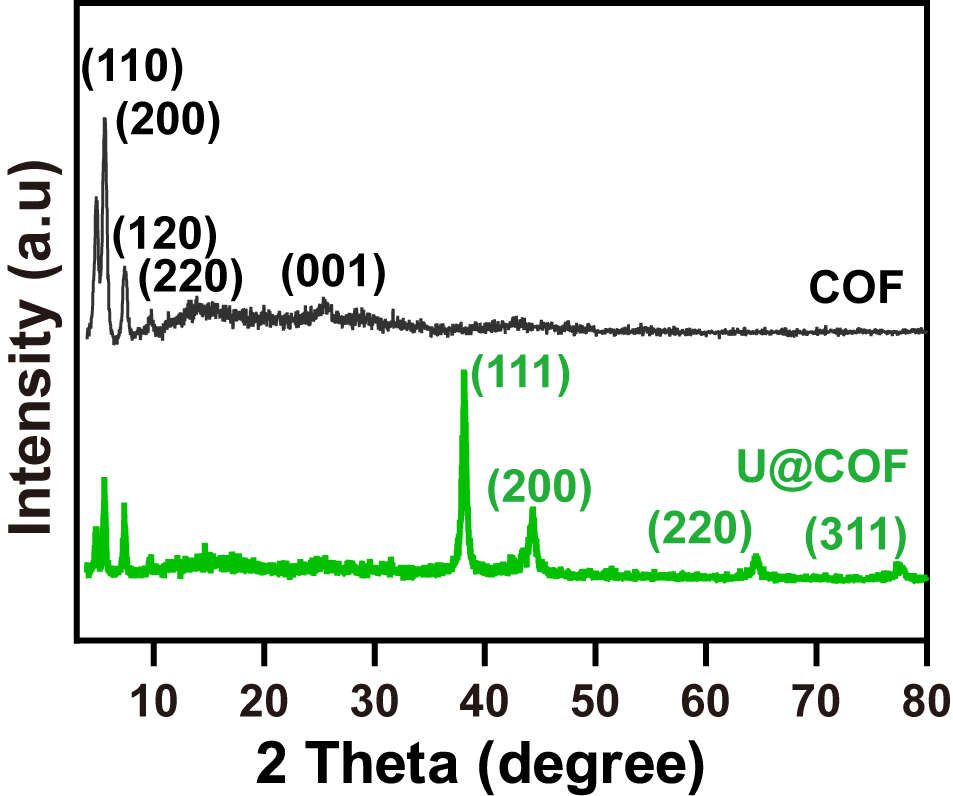


Figure S3. XRD pattern of COF and U@COF.


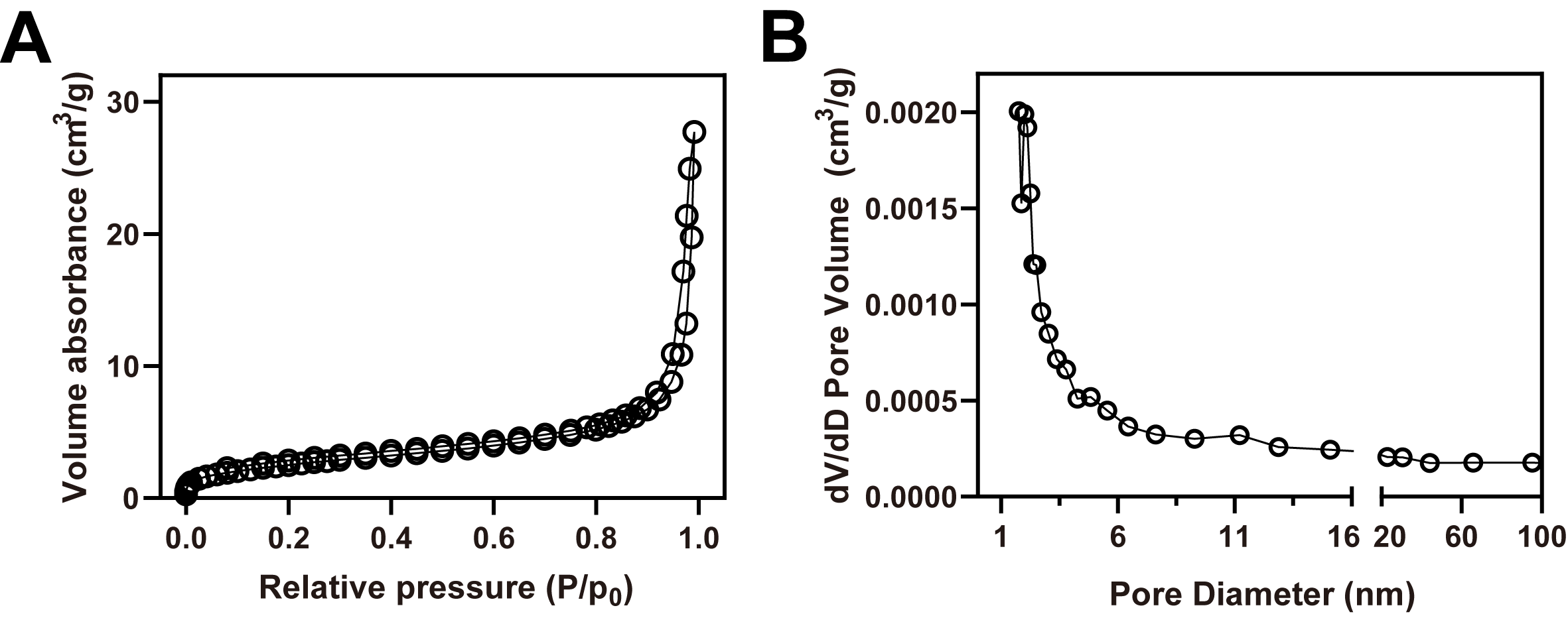


Figure S4. (A) N_2_ adsorption-desorption isotherms curve and (B) pore size distribution of U@COF.


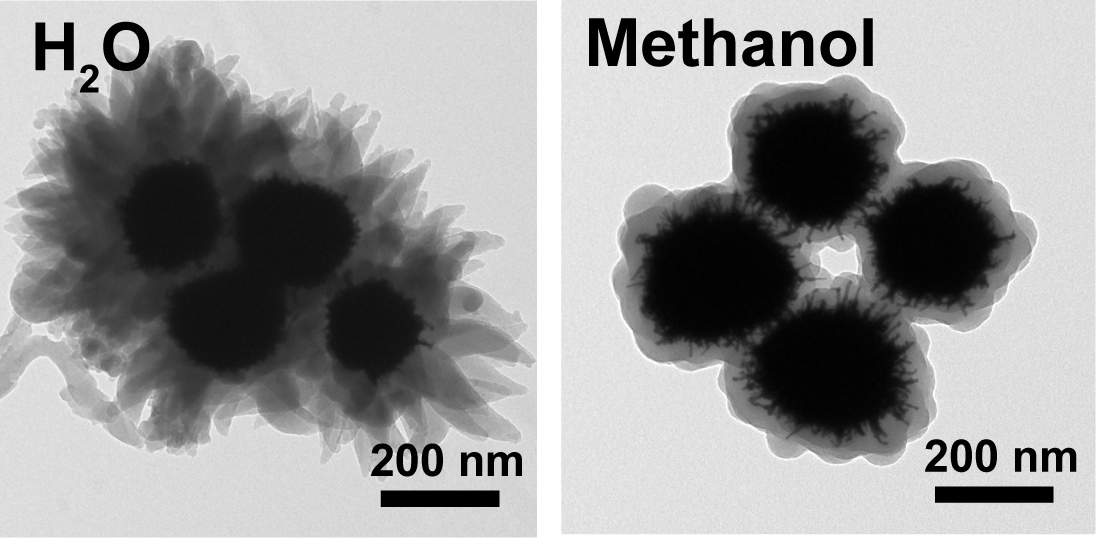


Figure S5. TEM images showing U@COF polymerization in different reaction solvent.


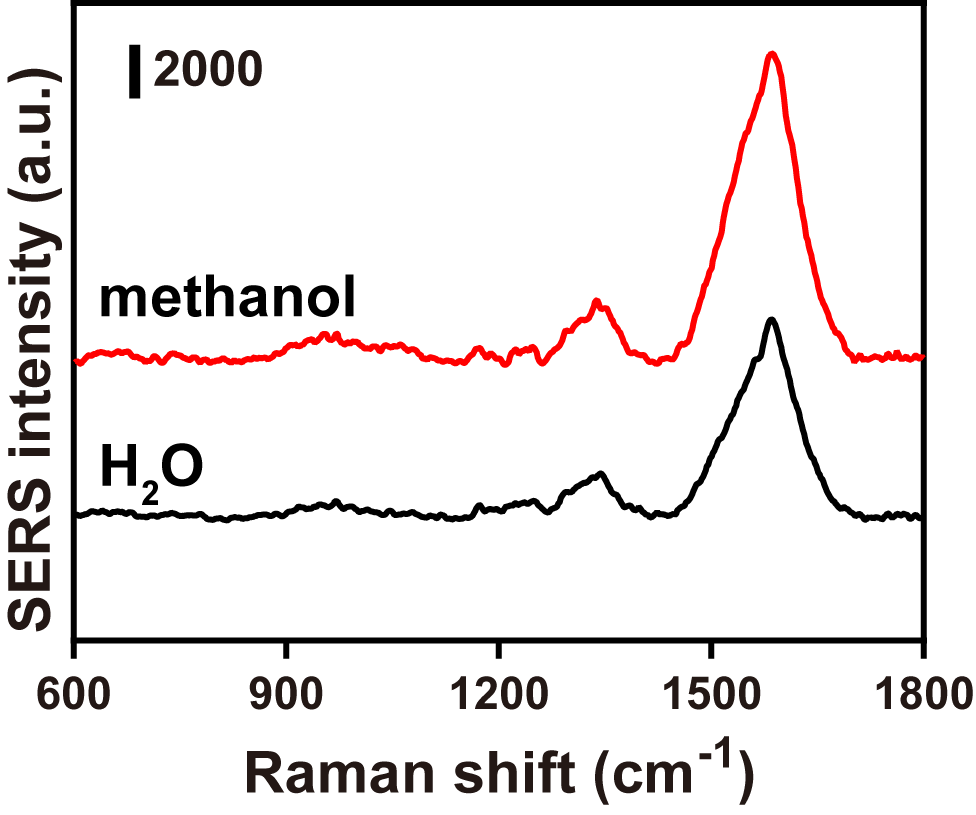


Figure S6. SERS spectra of U@COF polymerization in different reaction solvent.


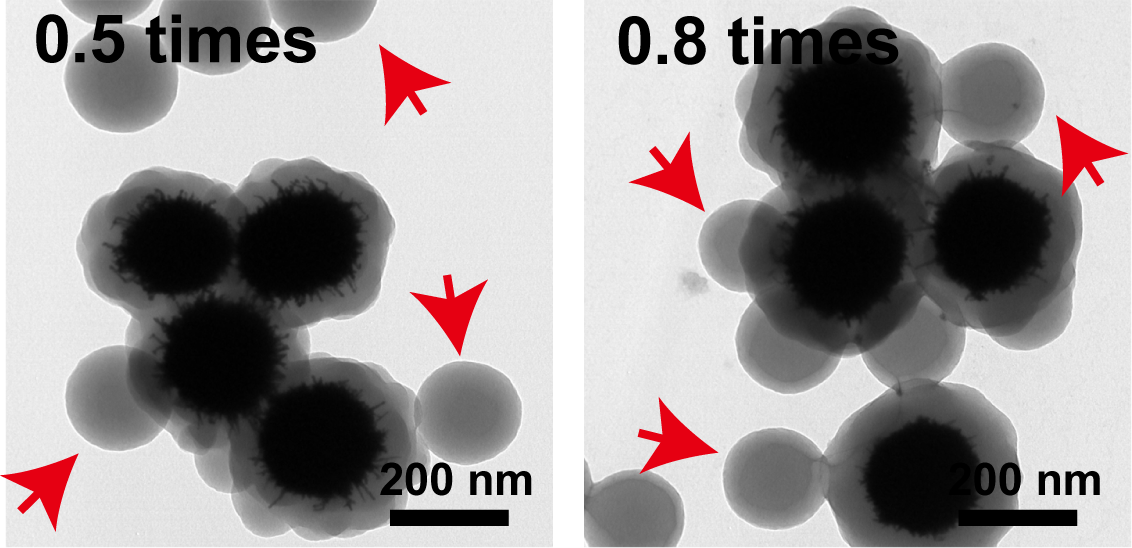


Figure S7. TEM images showing self-aggregation of COF precursors (See in the red arrows) surrounding around the U@COF.


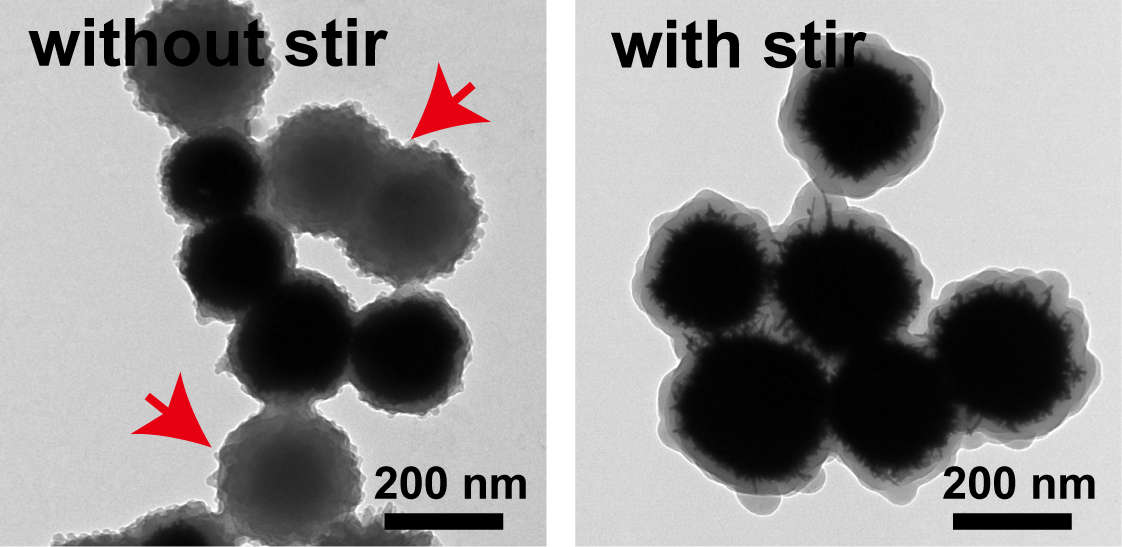


Figure S8. TEM images compared the dynamic and static synthesis of the U@COF. The red arrows indicated some rough COF structure as impurities.


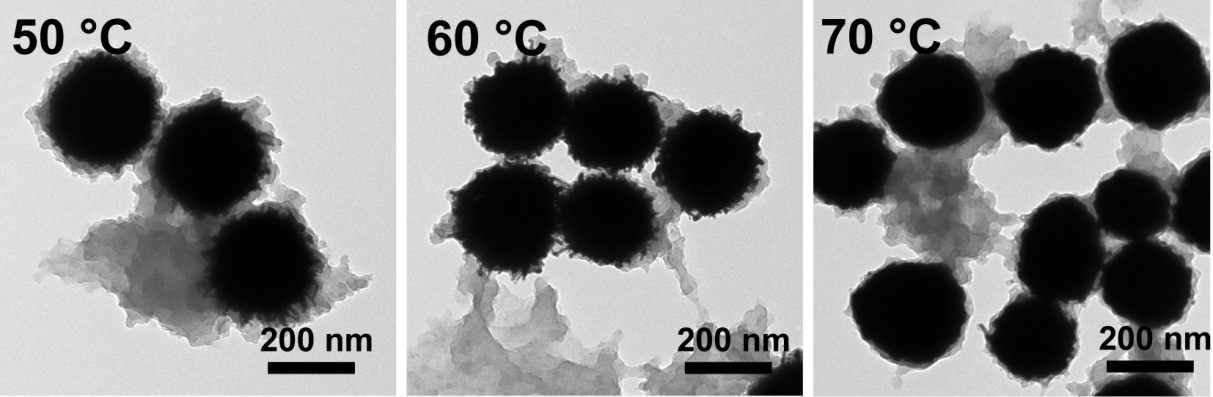


Figure S9. TEM images showing the polymerization of COF shell on U@COF at different temperature.


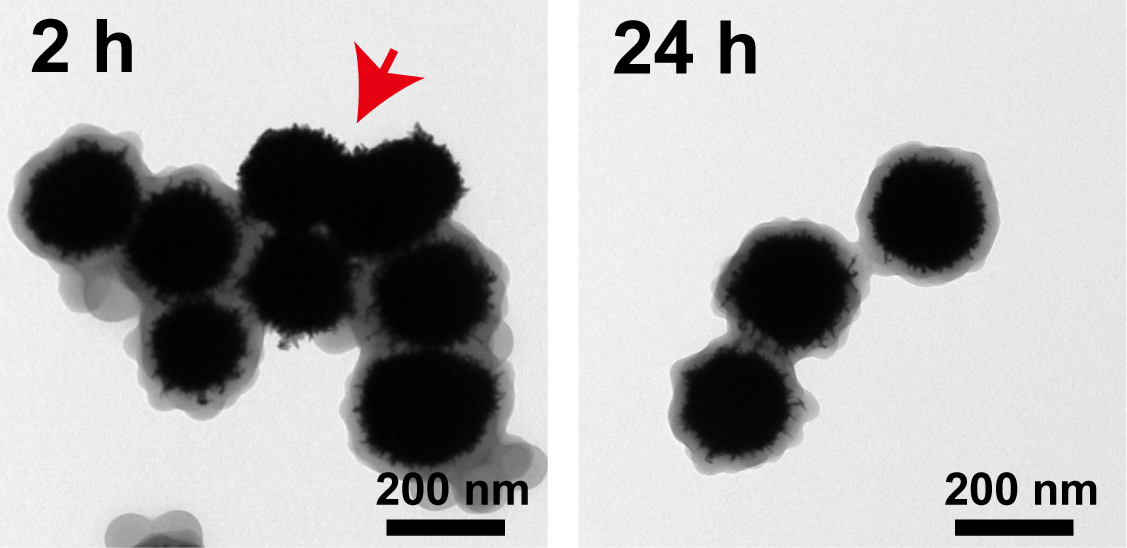


Figure S10. TEM images showing the polymerization of COF shell on U@COF at different time. The red arrows indicated the unencapsulated UAA.


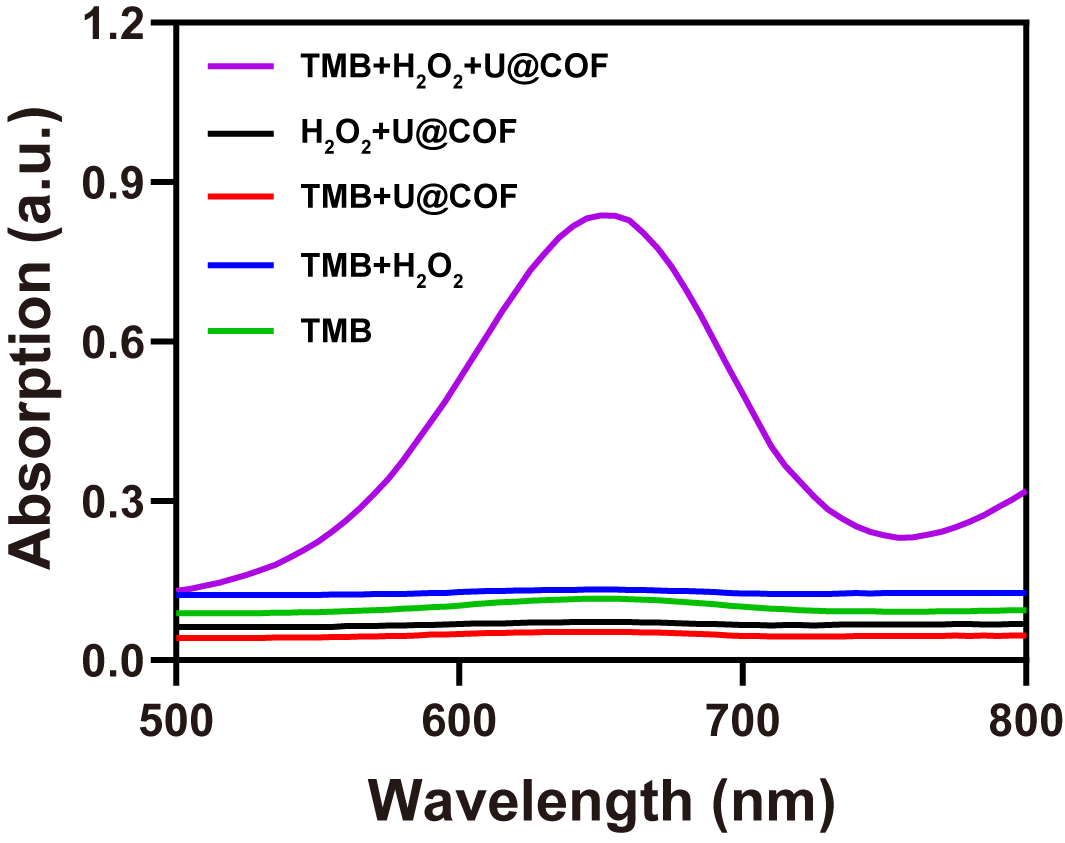


Figure S11. UV-vis absorption spectra of different treated groups. U@COF can only catalyze the oxidation of TMB with the assistance of H_2_O_2_, accompanied by the appearance of absorption at 652 nm and the change of color from colorless to blue.


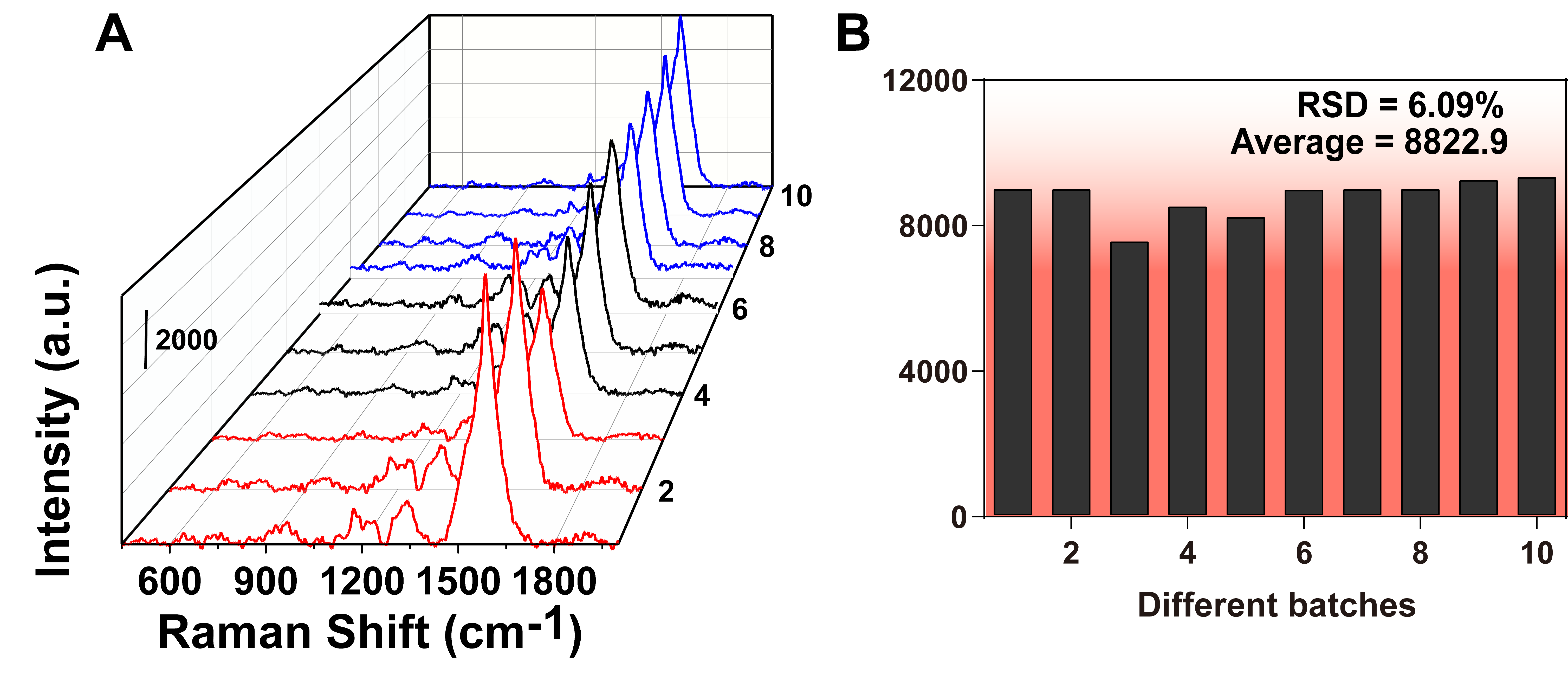


Figure S12. (A) SERS spectra and (B) the statistic results obtained from 10 batches of the U@COF_0.7_.


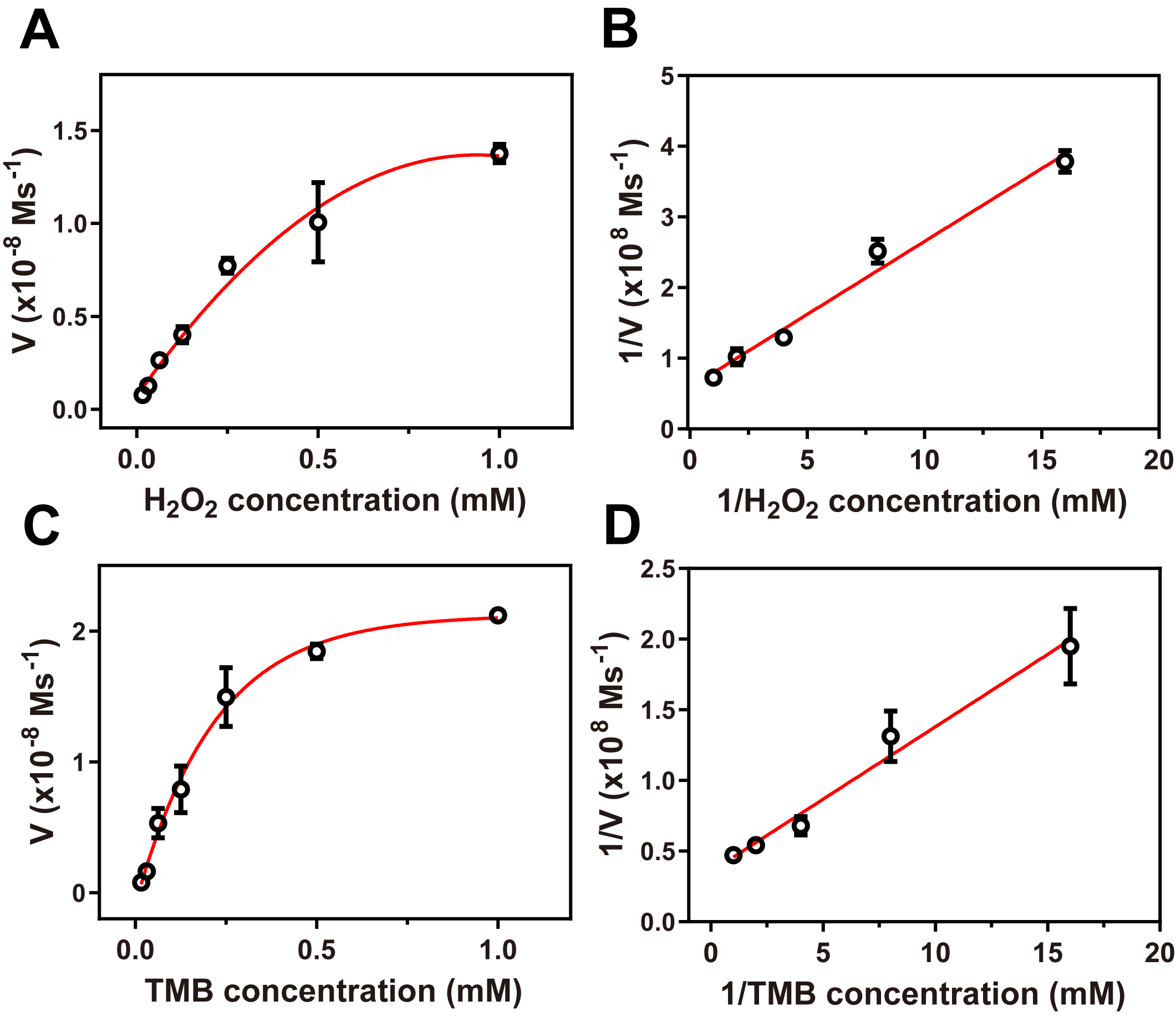


Figure S13. (A) Kinetics assays of U@COF with varying H_2_O_2_ concentration and fixed TMB concentration. (C) Kinetics assays of U@COF with varying TMB concentration and fixed H_2_O_2_ concentration. (B) and (D) were double-reciprocal plots of (A) and (C), respectively. Data are presented as mean ± SD. (*n=3*).


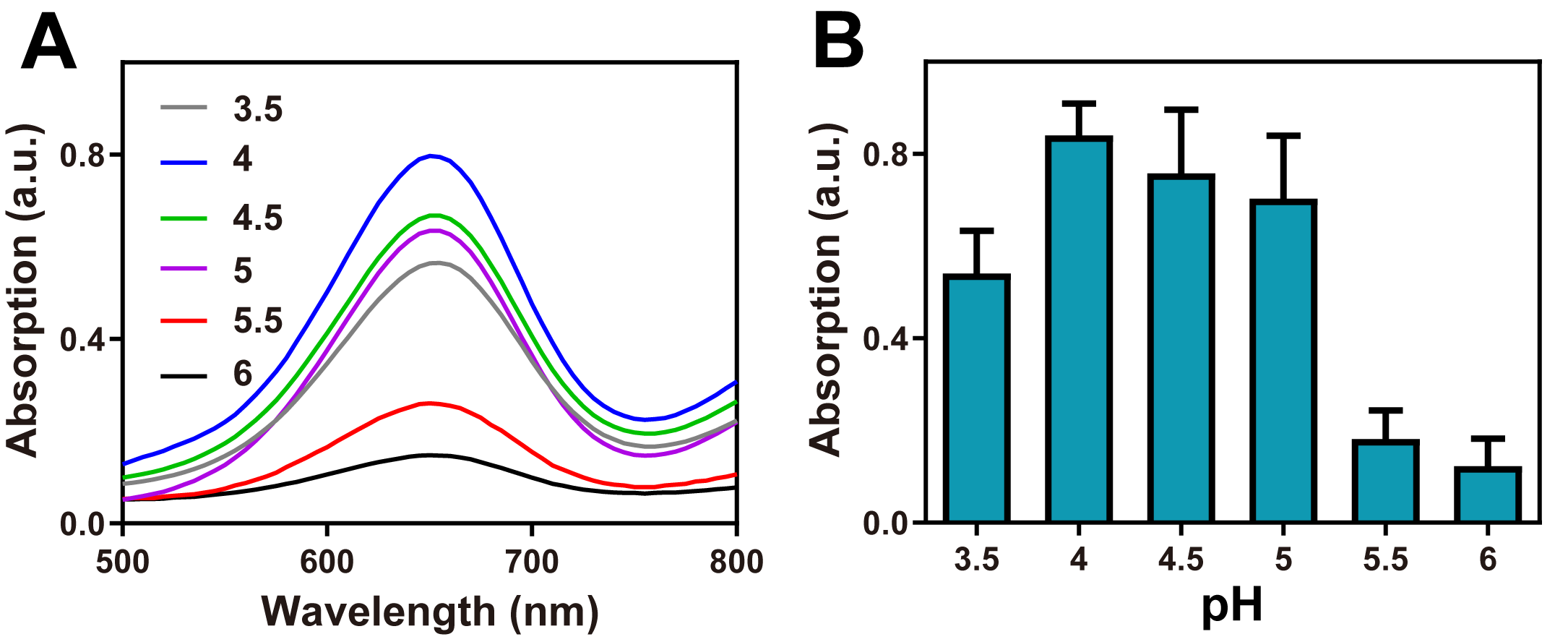


Figure S14. (A) UV-vis spectra for the oxidation reaction of TMB catalyzed by U@COF under the different pH. (B) UV-vis absorption intensity at 652 nm according to (A). Data are presented as mean ± SD. (*n=3*).


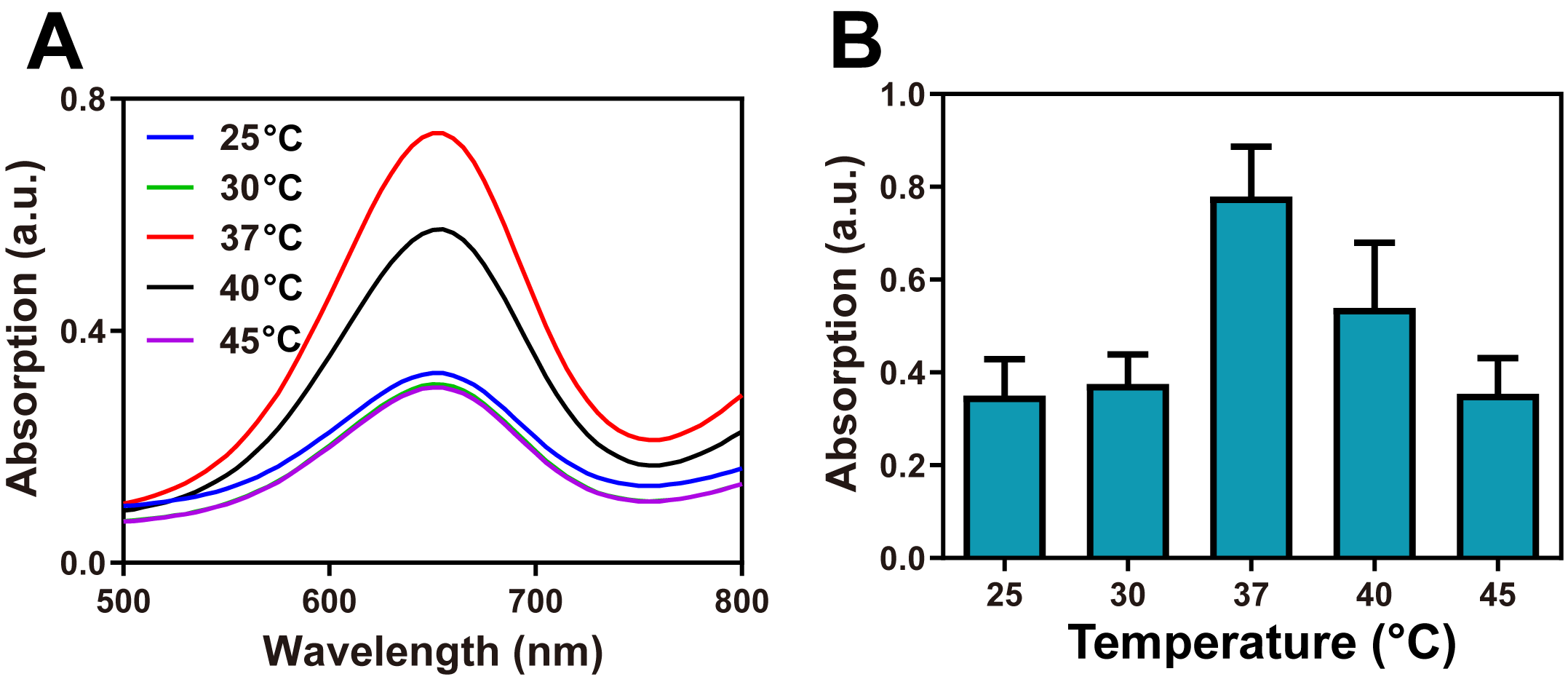


Figure S15. (A) UV-vis spectra for the oxidation reaction of TMB catalyzed by U@COF under the different temperature. (B) UV-vis absorption intensity at 652 nm according to (A). Data are presented as mean ± SD. (*n=3*).


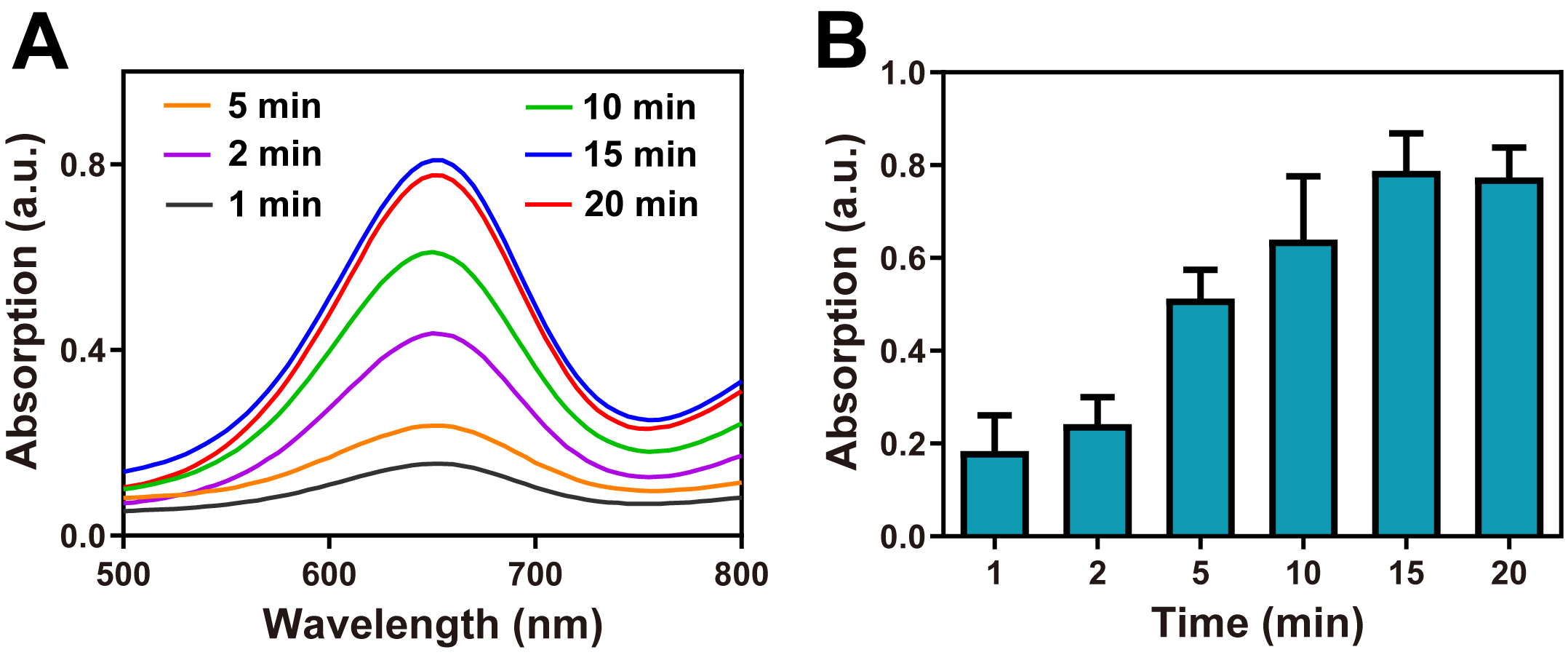


Figure S16. (A) UV-vis spectra for the oxidation reaction of TMB catalyzed by U@COF under the different time. (B) UV-vis absorption intensity at 652 nm according to (A). Data are presented as mean ± SD. (*n=3*).


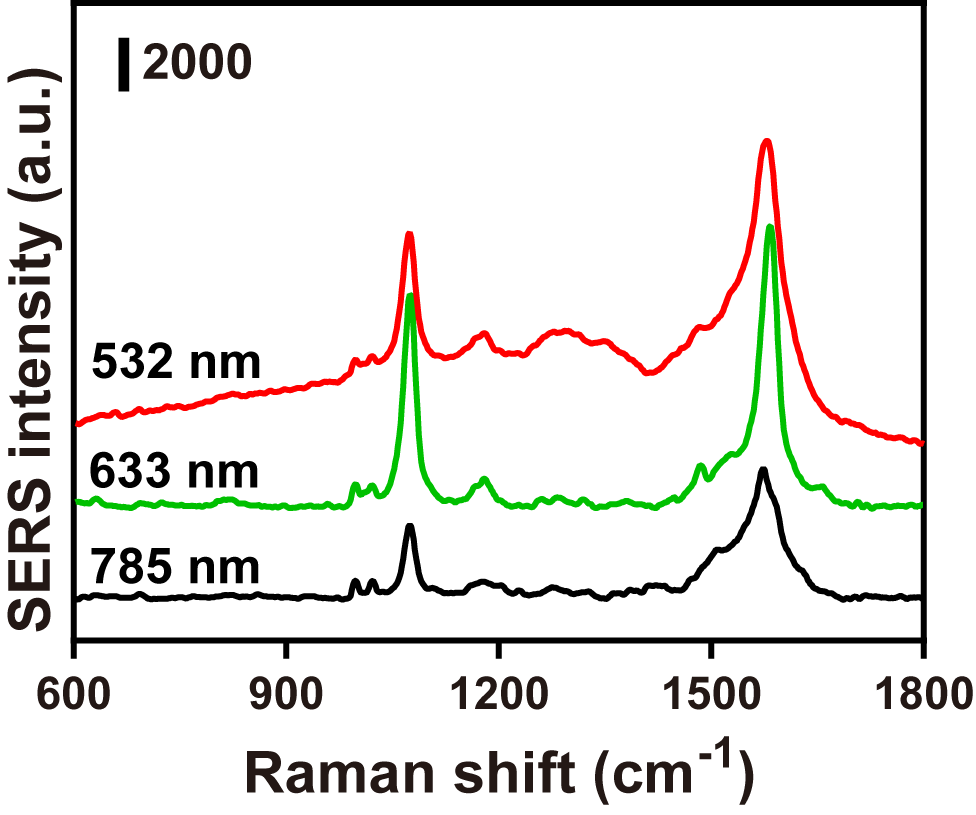


Figure S17. SERS spectra of nebulized 4-MPBA obtained under different excitation wavelengths (785 nm, 633 nm, 532 nm) with laser power at 1 mW.


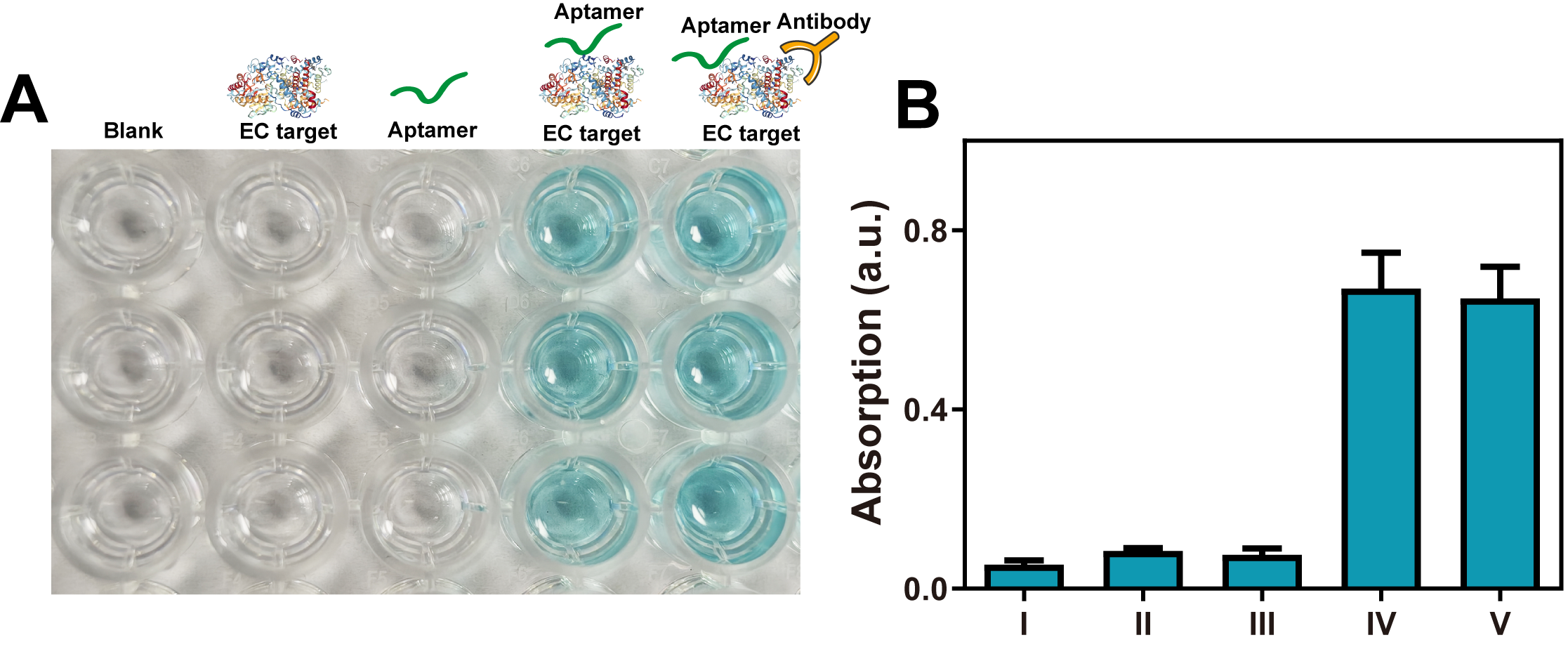


Figure S18. (A) Aptamer-antibody competition binding assay. (B) UV-vis absorption intensity at 652 nm according to (A). Data are presented as mean ± SD. I: blank, II: EC target, III: Apt-HRP, IV: EC target + Apt-HRP, V: EC target + Apt-HRP + antibody. All groups were added with TMB and catalyzed under H_2_O_2_.

**Note for** **the investigation of aptamer-antibody competition binding**

To further determine aptamer-antibody competition binding, the heterodimer EC target was coated onto a 96-well microtitre plate through physical adsorption, followed by adding the biotinylated aptamer and binding of HRP-conjugated streptavidin. The HRP linked on the aptamer (Apt-HRP) can catalyze 3,3’,5,5’- tetramethylbenzidine (TMB) molecules to oxidized TMB (oxTMB), with the color changes from colorless to blue. Obviously, only the Apt-HRP attached to the EC target can catalyze TMB into oxTMB. It is worth noting that there was no obvious color fading when the antibody and the aptamer were co-incubated with the EC target, indicating that the binding sites of aptamer was unaffected by the CFP-10 antibody. Since the different binding sites of antibody and aptamer, the two recognition modes are conducive to overcoming the problem of mutual interference of recognition sites and improving the recognition efficiency.


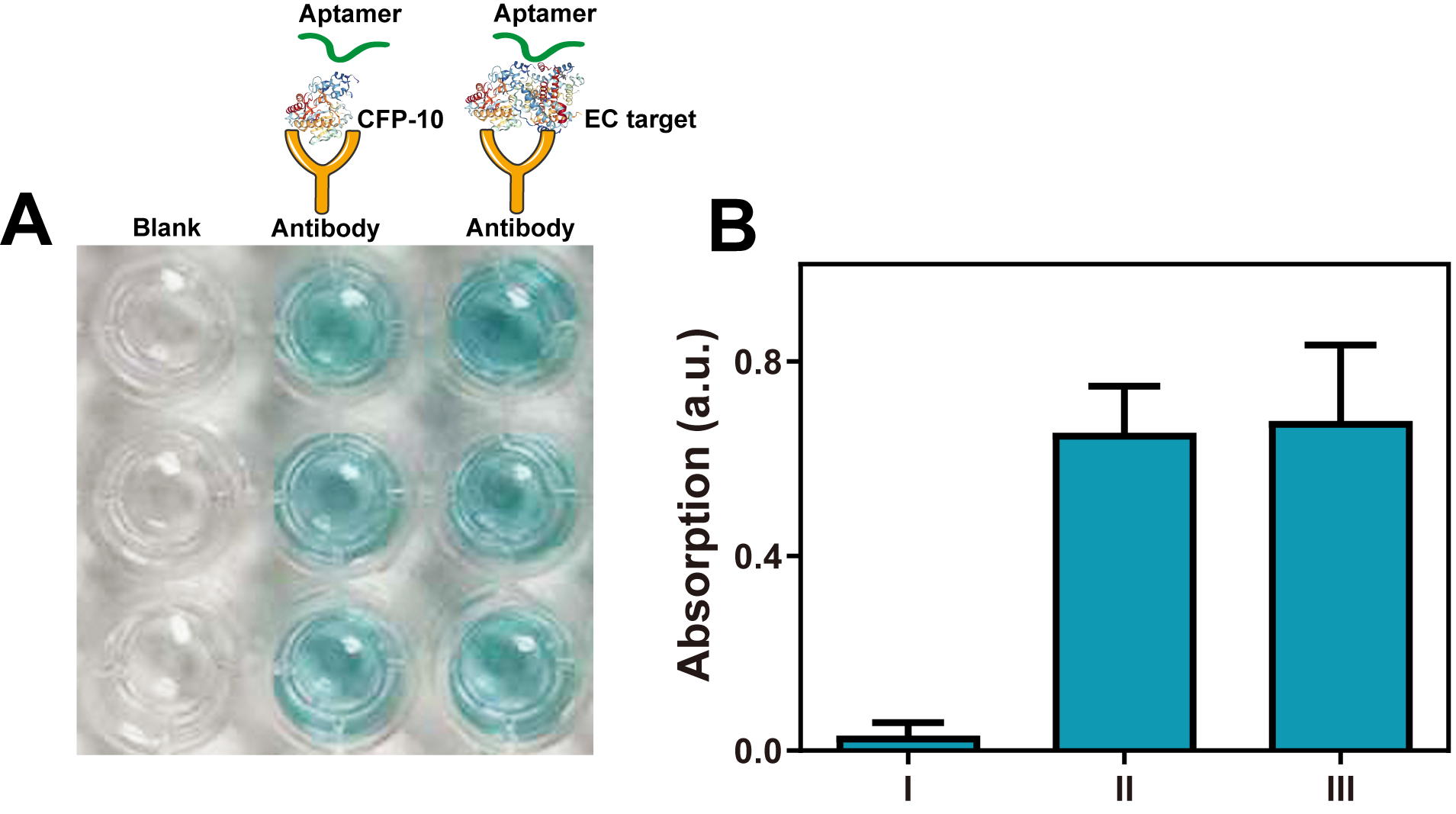


Figure S19. (A) EC heterodimers binding ability of CFP-10 antibodies assay. (B) UV-vis absorption intensity at 652 nm according to (A). Data are presented as mean ± SD. I: blank, II: antibody + CFP-10 + Apt-HRP, III: antibody + EC target + Apt-HRP. All groups were added with TMB and catalyzed under H_2_O_2_.

**Note for** **the investigation of EC heterodimers binding ability of CFP-10 antibodies**

To further determine EC heterodimers binding ability of CFP-10 antibodies, the CFP-10 antibody was incubated onto a 96-well microtitre plate overnight, followed by blocking by BSA and binding of the CFP10 monomer or CFP10/ESAT6 heterodimers. Then, the Apt-HRP was added, followed by catalyzing with TMB. The results indicated that since the CFP10 and ESAT6 can spontaneously form tight 1:1 heterodimer complexes under physiological conditions, the binding effect of CFP-10 antibody to CFP10/ESAT6 heterodimer is comparable to that of CFP10 monomer. These results confirmed the good binding ability of CFP-10 antibody for CFP10/ESAT6 heterodimer.


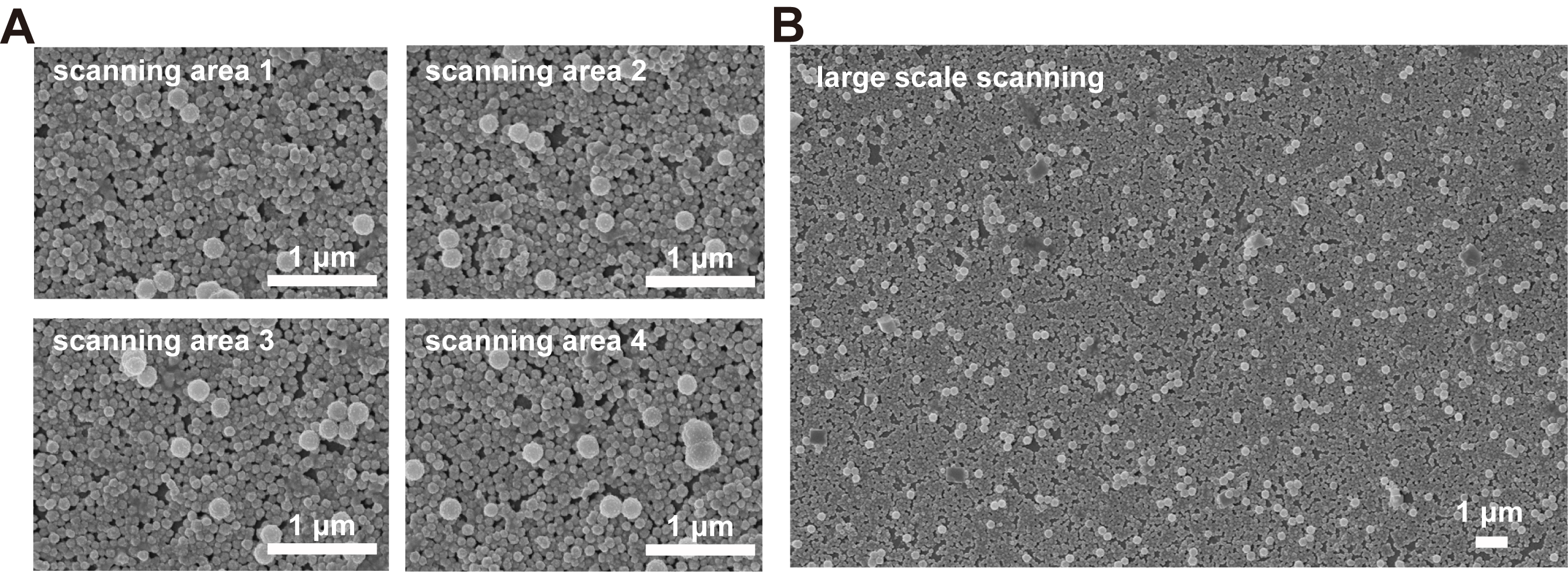


Figure S20. (A) SEM images of U@COF lingering on different area of Ag@AuNF array in the presence of ESAT-6/CFP-10. (B) SEM images of large scale scanning.


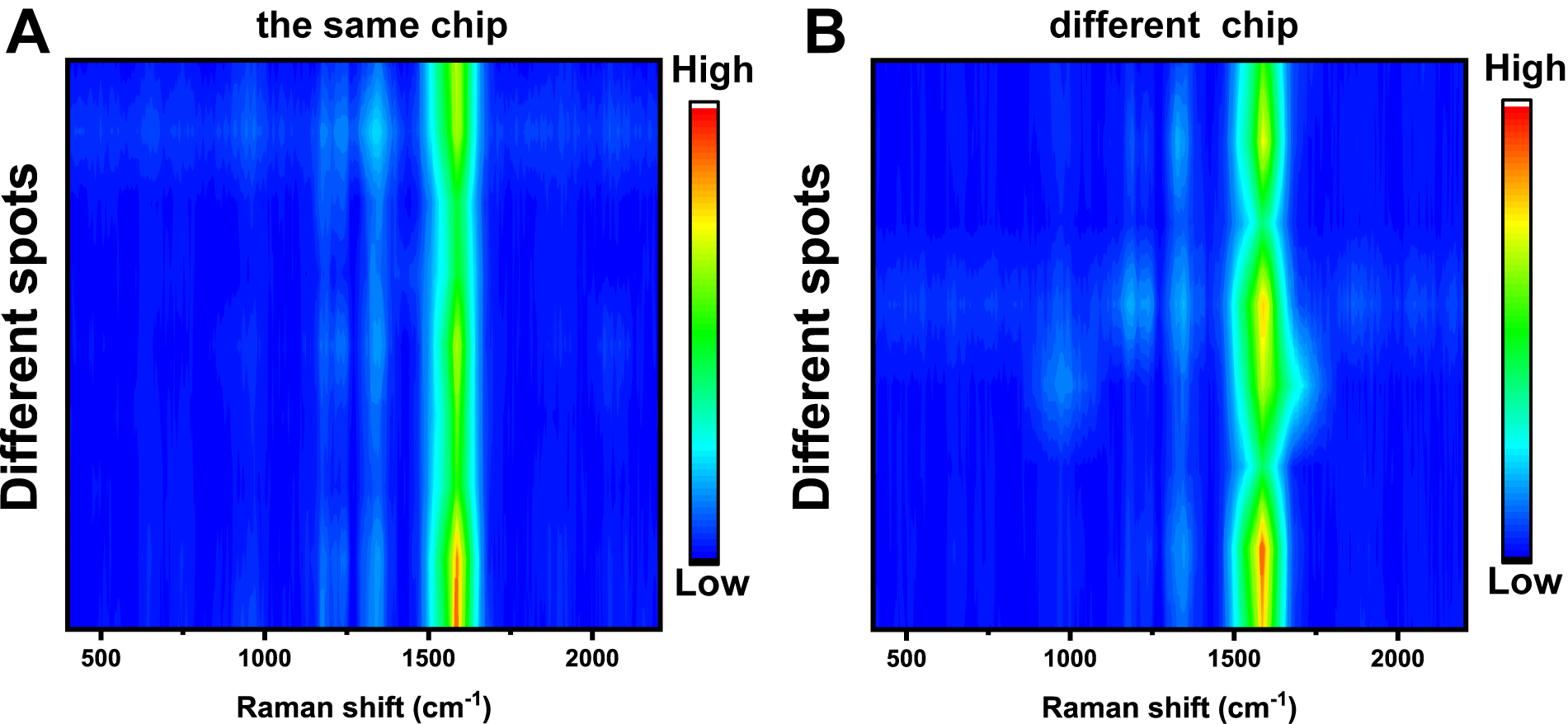


Figure S21. (A) 2D Raman spectra of different regions of the same chip (*n=30*). (B) 2D Raman spectra of different chip (*n=30*).


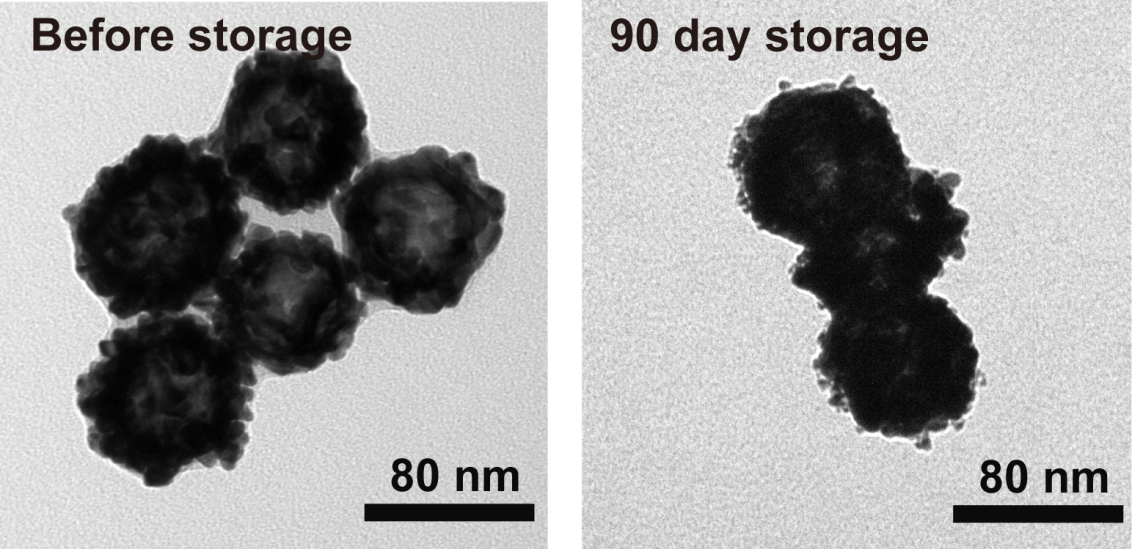


Figure S22. TEM images of Ag@AuNF stored for 90 days.


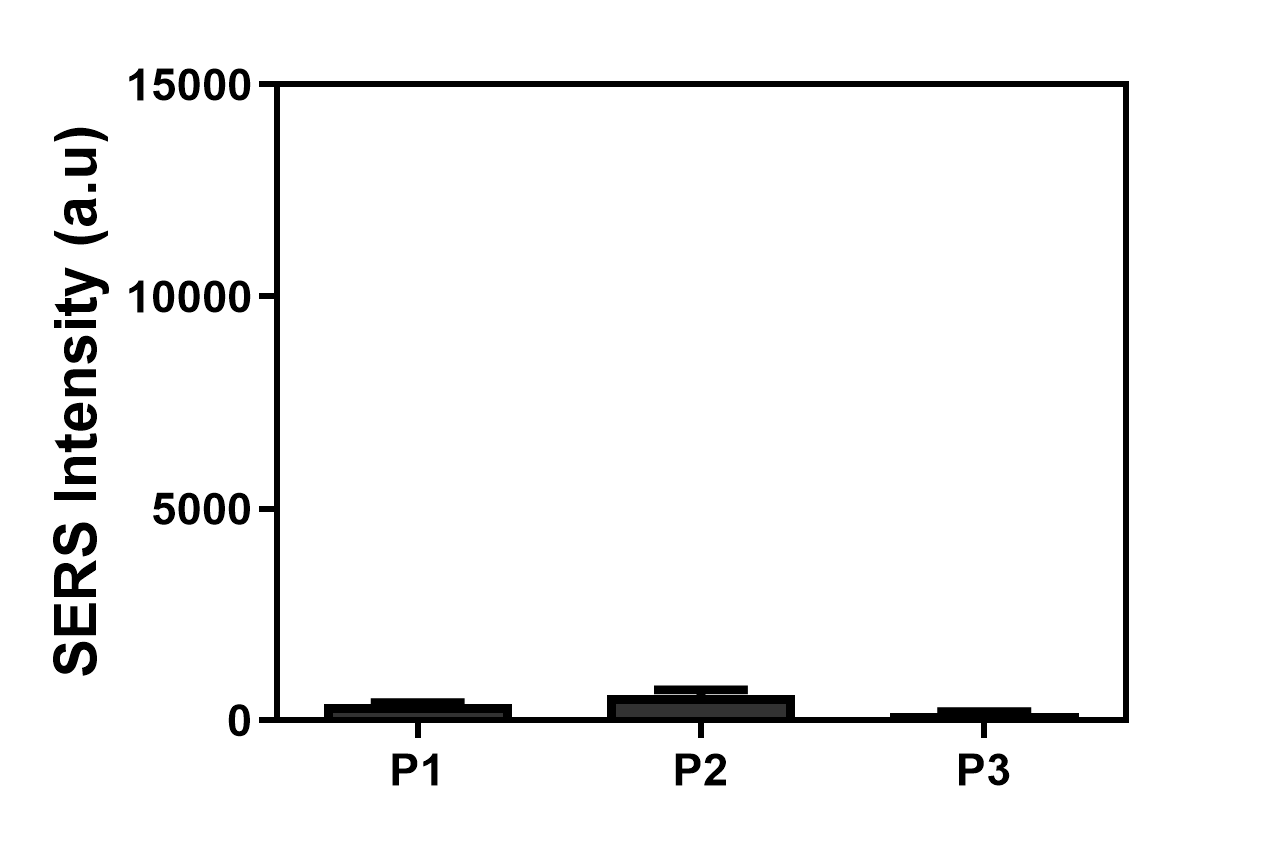


Figure S23. Detection of the ESAT-6/CFP-10 in exhaled gas from different patients. Data are presented as mean ± SD. (*n=3*).


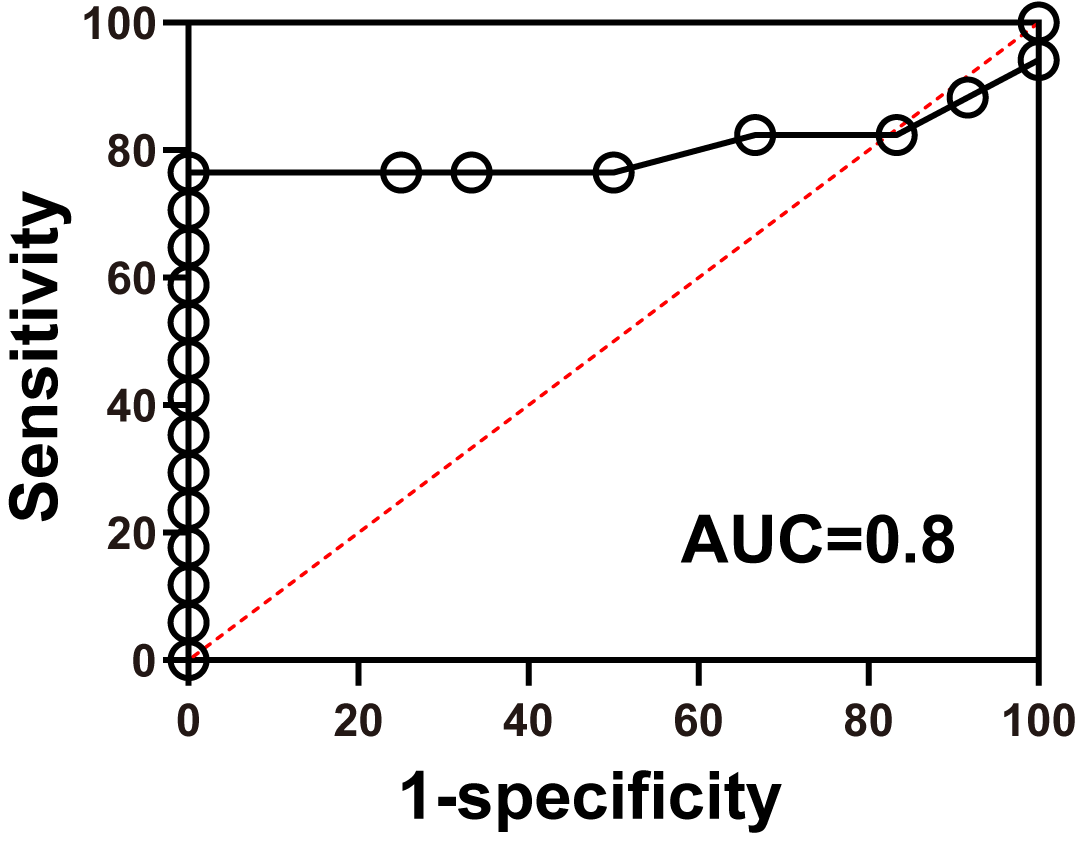


Figure S24. ROC curve of 29 clinical samples (negative (n=12), positive (n=17)). Area under the ROC curve is 0.8, *pROC < 0.05*, threshold is 0. 7485 ng/mL, sensitivity is 76.5%, and specificity is 100%.

Table S1. Comparison of CFP-10/ESAT-6 detection in different methods

| Platform | Linear ranges | LOD | Specimen | Advantage | Reference |
| --- | --- | --- | --- | --- | --- |
| Electrochemical immunosensor | 10–500  ng/mL | 1.5  ng/mL | Sputum | Rapid, simple, detection time < 2 h | [1] |
| Electrochemical biosensor | 5–500  ng/mL | 1.5 ng/mL | Sputum | High specificity, good reproducibility | [2] |
| Aptamer fluorescence testing | 0.01 ag/mL–1 μg/mL | 0.1 ag/mL | Blood | Rapid, high sensitivity and specificity | [3] |
| MSPQC sensor | / | ≤10³ CFU/mL | / | Highly specificity, low cost, easy operation, detection time 96.3 h | [4] |
| Impedimetric immunosensor | 0.5–50  ng/mL | 4.80  ng/mL | / | small sample volume | [5] |
| Nanopore amplification mechanisms | 100 aM–1 nM | 10 aM | Blood | nonsputum-based feature, low-volume sample | [6] |
| SERS/colorimetric mask | 2–200 ng/mL for SERS  5–200  ng/mL for colorimetry | 0.437 ng/mL | Droplets or expectoration | Portable, easy to operate, highly sensitive | This work |

Table S2. Recovery rate of ESAT-6/CFP-10 detection in sputum samples. (*n=3*).

| Sample  no | Spiked concentration  EC (ng/mL) | Detected concentration  LgC (ng/mL) | | Recovery  (%) | | RSD  (%) | |
| --- | --- | --- | --- | --- | --- | --- | --- |
|  |  | SERS | colorimetry | SERS | colorimetry | SERS | colorimetry |
| 1 | 20 | 21.3 | 18.9 | 106.5 | 94.5 | 4.0 | 3.8 |
| 2 | 50 | 48.5 | 45.6 | 97 | 91.2 | 7.2 | 9.2 |
| 3 | 100 | 89.5 | 95.6 | 89.5 | 95.6 | 8.3 | 10.2 |

Table S3. Clinical background of non-TB patients and the results of ESAT-6/CFP-10 assay.

| Sample  no | Sex | age | SERS-droplet test | |
| --- | --- | --- | --- | --- |
|  |  |  | EC level  (ng/mL) | SD |
| non-TB1 | M | 32 | 0.13 | 0.13 |
| non-TB2 | M | 44 | 0.11 | 0.11 |
| non-TB3 | M | 62 | 0.15 | 0.15 |
| non-TB4 | M | 88 | 0.15 | 0.15 |
| non-TB5 | F | 37 | 0.16 | 0.16 |
| non-TB6 | M | 36 | 0.18 | 0.18 |
| non-TB7 | M | 39 | 0.18 | 0.05 |
| non-TB8 | F | 42 | 0.18 | 0.07 |
| non-TB9 | F | 34 | 0.14 | 0.05 |
| non-TB10 | F | 59 | 0.16 | 0.03 |
| non-TB11 | F | 35 | 0.17 | 0.02 |
| non-TB12 | F | 38 | 0.14 | 0.08 |

Notice: *N.A.*= not applicable. Three replicates (*n = 3*) were run for each test.

Table S4. Clinical background of TB patients and the results of ESAT-6/CFP-10 assay.

| Sample  no | Sex | age | SERS-droplet test | |
| --- | --- | --- | --- | --- |
|  |  |  | EC level  (ng/mL) | SD |
| TB1 | M | 32 | 0.13 | 0.09 |
| TB2 | F | 28 | 0.15 | 0.08 |
| TB3 | F | 51 | 0.11 | 0.07 |
| TB4 | F | 48 | 52.50 | 11.66 |
| TB5 | F | 62 | 0.10 | 0.04 |
| TB6 | M | 29 | 3.67 | 1.36 |
| TB7 | F | 37 | 57.80 | 5.40 |
| TB8 | F | 66 | 48.27 | 7.81 |
| TB9 | M | 68 | 1.32 | 0.66 |
| TB10 | M | 72 | 45.07 | 8.75 |
| TB11 | M | 36 | 3.35 | 1.05 |
| TB12 | M | 37 | 6.69 | 1.94 |
| TB13 | M | 39 | 10.07 | 3.86 |
| TB14 | F | 58 | 48.45 | 5.64 |
| TB15 | F | 40 | 50.17 | 4.08 |
| TB16 | F | 48 | 7.88 | 1.68 |
| TB17 | F | 36 | 8.13 | 2.72 |

Notice: *N.A.*= not applicable. Three replicates (*n = 3*) were run for each test.

Table S5. Clinical background of Close contacts and the results of ESAT-6/CFP-10 assay.

| Sample  no | Sex | age | symptom | SERS-droplet test | |
| --- | --- | --- | --- | --- | --- |
|  |  |  |  | EC level  (ng/mL) | SD |
| Close contact TB1 | F | 32 | *N.A.* | 0.05 | 0.04 |
| Close contact TB2 | F | 35 | × | 0.07 | 0.05 |
| Close contact TB3 | M | 51 | √ | 19.77 | 1.88 |
| Close contact TB4 | M | 27 | × | 0.05 | 0.07 |
| Close contact TB5 | M | 58 | × | 0.13 | 0.09 |
| Close contact TB6 | M | 62 | × | 0.17 | 0.15 |

Notice: *N.A.*= not applicable. Three replicates (*n = 3*) were run for each test.

**References**

[1] U. Z. M. Azmi, N. A. Yusof, J. Abdullah, S. A. A. Ahmad, F. N. M. Faudzi, N. H. A. Raston, S. Suraiya, P. S. Ong, D. Krishnan, N. K. Sahar, Portable electrochemical immunosensor for detection of mycobacterium tuberculosis secreted protein CFP10-ESAT6 in clinical sputum samples, *Mikrochimica Acta* **2021**, *188 (1)*, 20.

[2] U. Z. M.Azmi, N. A. Yusof, J. Abdullah, F. Mohammad, S. A. A. Ahmad, S. Suraiya, N. H. A. Raston, F. N. M. Faudzi, S. K. Khiste, H. A A.-Lohedan, Aptasensor for the Detection of Mycobacterium tuberculosis in Sputum Utilising CFP10-ESAT6 Protein as a Selective Biomarker, *Nanomaterials* **2021**, *11 (9)*, 2446.

[3] S. Liu, G. Xiao, P. Li, Y. Xu, X. Fan, L. Tian, S. Zhang, G. Zhang, Plasma-based ultrasensitive detection of Mycobacterium tuberculosis ESAT6/CFP10 fusion antigen using a CRISPR-driven aptamer fluorescence testing (CRAFT), *Biosensors & bioelectronics* **2025**, *284*, 117566.

[4] F. He, Y. Xiong, J. Liu, F. Tong, D. Yan, Construction of Au-IDE/CFP10-ESAT6 aptamer/DNA-AuNPs MSPQC for rapid detection of Mycobacterium tuberculosis, *Biosensors & bioelectronics* **2016**, *77*, 799.

[5] X. Wang, X. Wei, M. M. v. d. Zalm, Z. Zhang, N. Subramanian, A.-M. Demers, E. G. Walters, A. Hesseling, C. Liu, Quantitation of Circulating Mycobacterium tuberculosis Antigens by Nanopore Biosensing in Children Evaluated for Pulmonary Tuberculosis in South Africa, ACS Nano **2023**, *17(21)*, 21093.

[6] N. M. Bakhori, N. A. Yusof, J. Abdullah, H. Wasoh, S. K. A. Rahman, S. F. A. Rahman, Surface Enhanced CdSe/ZnS QD/SiNP Electrochemical Immunosensor for the Detection of Mycobacterium Tuberculosis by Combination of CFP10-ESAT6 for Better Diagnostic Specificity, *Materials* **2019**, *13(1)*, 149.
